# Whole-Transcriptome Analysis Reveals Autophagy Is Involved in Early Senescence of *zj-es* Mutant Rice

**DOI:** 10.3389/fpls.2022.899054

**Published:** 2022-06-03

**Authors:** Jia Sun, Weifang Liang, Shenghai Ye, Xinyu Chen, Yuhang Zhou, Jianfei Lu, Ying Shen, Xuming Wang, Jie Zhou, Chulang Yu, Chengqi Yan, Bingsong Zheng, Jianping Chen, Yong Yang

**Affiliations:** ^1^College of Life Science, Fujian A&F University, Fuzhou, China; ^2^State Key Laboratory for Managing Biotic and Chemical Threats to the Quality and Safety of Agro-Products, Key Laboratory of Biotechnology for Plant Protection, Ministry of Agriculture, and Rural Affairs, Zhejiang Provincial Key Laboratory of Biotechnology for Plant Protection, Institute of Virology and Biotechnology, Zhejiang Academy of Agricultural Science, Hangzhou, China; ^3^College of Plant Protection, Yunnan Agricultural University, Kunming, China; ^4^Institute of Crop and Nuclear Technology Utilization, Zhejiang Academy of Agricultural Sciences, Hangzhou, China; ^5^College of Plant Protection, Shenyang Agricultural University, Shenyang, China; ^6^State Key Laboratory of Subtropical Silviculture, Zhejiang A&F University, Hangzhou, China; ^7^Zhejiang Plant Protection, Quarantine and Pesticide Management Station, Hangzhou, China; ^8^State Key Laboratory for Managing Biotic and Chemical Threats to the Quality and Safety of Agro-Products, Key Laboratory of Biotechnology for Plant Protection, Ministry of Agriculture, and Rural affairs, Zhejiang Provincial Key Laboratory of Biotechnology for Plant Protection, Institute of Plant Virology, Ningbo University, Ningbo, China; ^9^Institute of Biotechnology, Ningbo Academy of Agricultural Science, Ningbo, China

**Keywords:** whole-transcriptome, autophagy, ROS, cell death, early senescence

## Abstract

Senescence is a necessary stage of plant growth and development, and the early senescence of rice will lead to yield reduction and quality decline. However, the mechanisms of rice senescence remain obscure. In this study, we characterized an early-senescence rice mutant, designated *zj-es* (*ZheJing-early senescence*), which was derived from the japonica rice cultivar Zhejing22. The mutant *zj-es* exhibited obvious early-senescence phenotype, such as collapsed chloroplast, lesions in leaves, declined fertility, plant dwarf, and decreased agronomic traits. The *ZJ-ES* gene was mapped in a 458 kb-interval between the molecular markers RM5992 and RM5813 on Chromosome 3, and analysis suggested that *ZJ-ES* is a novel gene controlling rice early senescence. Subsequently, whole-transcriptome RNA sequencing was performed on *zj-es* and its wild-type rice to dissect the underlying molecular mechanism for early senescence. Totally, 10,085 differentially expressed mRNAs (DEmRNAs), 1,253 differentially expressed lncRNAs (DElncRNAs), and 614 differentially expressed miRNAs (DEmiRNAs) were identified, respectively, in different comparison groups. Based on the weighted gene co-expression network analysis (WGCNA), the co-expression turquoise module was found to be the key for the occurrence of rice early senescence. Furthermore, analysis on the competing endogenous RNA (CeRNA) network revealed that 14 lncRNAs possibly regulated 16 co-expressed mRNAs through 8 miRNAs, and enrichment analysis showed that most of the DEmRNAs and the targets of DElncRNAs and DEmiRNAs were involved in reactive oxygen species (ROS)-triggered autophagy-related pathways. Further analysis showed that, in *zj-es*, ROS-related enzyme activities were markedly changed, ROS were largely accumulated, autophagosomes were obviously observed, cell death was significantly detected, and lesions were notably appeared in leaves. Totally, combining our results here and the remaining research, we infer that ROS-triggered autophagy induces the programmed cell death (PCD) and its coupled early senescence in *zj-es* mutant rice.

## Introduction

Plant senescence is an age-dependent degradation process at the level of cells, tissues, organs, or organisms, leading to death or end of life (Lim et al., 2007). The obvious indicator of plant senescence is the gradual yellowing of leaves, which often starts from the leaf tip downward, toward the veins from interveinal regions of the lamina (Thomas, 2013). Concomitant with the alteration on leaf color, plants during senescence will also undergo some other internal changes, such as on cell/sub-cell structure, physiological metabolisms, and biochemical reactions (Finkelstein and Rock, 2002; Hirayama and Shinozaki, 2007; Robert-Seilaniantz et al., 2011). For example, chloroplast is often the first target to be dismantled to produce chlorophyll catabolites, and the grana structure with its contents in chloroplasts is also changed (Hudson et al., 2013; Yamatani et al., 2013). Meanwhile, the content of polysomes and ribosomes decreased, and the plasma and vacuolar membranes are gradually collapsed (Evans et al., 2010). The decline in photosynthetic activity is also one of the early indicators of senescence, and senescence influences the photosynthetic electron transport chain by inducing the changes in the protein composition of the photosynthetic apparatus (Schottler et al., 2017; Krieger-Liszkay et al., 2019). In addition, as plant senescence occurs, anabolic processes, such as assimilation of nitrogen, phosphorus, and potassium, are reduced, and the catabolism of proteins, lipids, and nucleic acids becomes prominent (Lim et al., 2007).

One of the most notable features of plant senescence is the accumulation of reactive oxygen species (ROS), including superoxide anion radical (O⋅^–2^), hydroxyl free radicals (OH⋅), and hydrogen peroxide (H_2_O_2_) (Balazadeh et al., 2012). The ROS status has been found to be controlled by a fine-tuned network of enzymatic and antioxidative components, consisting of ROS-producing and ROS-scavenging proteins, and then plant cellular ROS concentration increases rapidly if the equilibration between its production and scavenging is perturbed (Mittler et al., 2004). Numerous studies have revealed that the crucial and complicated process of plant senescence is highly influenced by ROS production (Woo et al., 2013), for instance, senescence in the *OsGDCH*-RNAi rice plants is induced by excessive accumulation of ROS (Zhou et al., 2013). One of the roles that ROS play in plant senescence is considered that ROS leads to the protein and lipid oxidation and dysfunction, which are collectively referred to as senescence-associated syndromes (Vanacker et al., 2006). ROS have also been widely proved as a kind of signal molecules to initiate some signals transduction that triggers plant senescence. The expressions of many senescence-associated genes (SAGs) in Arabidopsis, such as *LSC54*, *LSC94*, *LSC760*, *LSC790*, and *LSC803*, were also enhanced by increased ROS levels. Bieker et al. (2012) have found that, in oilseed rape and Arabidopsis, the H_2_O_2_ level increased during bolting and flowering time but did not increase at the late senescence stage. And, under elevated CO_2_ conditions, H_2_O_2_ content increased in advance with the accelerated senescence rate of plants, suggesting that H_2_O_2_ may be one of the signals of inducing plant senescence (Smykowski et al., 2010; Bieker et al., 2012).

Senescence is the final stage of plant growth and development, followed by the death of a wide range of cells, tissues, and organs, which is associated with an increase in peroxides, such as H_2_O_2_, and O⋅^–2^. Senescence can be inhibited by ethylene antagonists and cytokinins. All of these observations suggest that the senescence process involves signaling pathways (Petrov et al., 2015; Wang et al., 2019b; Gao et al., 2021). So some scholars believe that plant senescence is related to programmed cell death (PCD). In addition, during early senescence, senescence-related genes encoding cysteine proteases were found in Arabidopsis and tomato, which is similar to an enzyme involved in animal PCD, suggesting that they play a role in PCD in plant senescence. During later senescence, epidermal cells undergo PCD, with the simultaneous upregulation of *LoSAG12* (Drake et al., 1996; Mochizuki-Kawai et al., 2015; Buono et al., 2019). Recently, overexpression of PCD-related gene *BnaNAC60* in both tobacco and oilseed rape protoplasts could induce significant ROS accumulation and hypersensitive response-like cell death, ultimately resulting in leaf senescence, which provides new evidence for the relationship between plant senescence and PCD (Yan et al., 2021).

Plant senescence is a highly regulated process accompanied by changes in gene expression, and many senescence-related genes in plants have been identified. These genes are generally divided into several categories depending on their metabolic pathways. One class of genes is involved in chloroplast synthesis and chlorophyll degradation. During senescence, mass chlorophyll degradation was induced by the co-upregulated expression of *SGR* and *PaO* in plant leaves (Jiang et al., 2007). *NYC1* and *NOL* encode chlorophyll *b* reductases with divergent functions, thereby mediating the process of plant senescence (Kusaba et al., 2007). *V1* encodes a plastid protein NUS1, which is involved in the regulation of chloroplast RNA metabolism and promotes the establishment of the plastid genetic system during early chloroplast development (Kusumi et al., 2011). The second type of genes is involved in hormone signaling pathways. For example, ABA-promoted expression of *OsNAP* directly upregulates genes, such as *SGR*, *NYC1*, and *RCCR1*, leading to early leaf senescence (Liang et al., 2014). Some senescence-related genes, including *EIN2*, *EIN3*, *OsFBK12*, and *OsSAMS1*, are involved in ethylene signaling pathways (Alonso et al., 1999; Chen et al., 2013; Li et al., 2013). The third category is genes related to protein synthesis, degradation, and transport. For instance, *TDC1* plays a major role in serotonin biosynthesis during plants senescence (Kang et al., 2009). *Osh69* is located in the chloroplasts of senescent leaves, and its expression level appears to be upregulated when plants are challenged with senescence (Lee et al., 2004). *OsSAG12-1* encodes a protease, which is mainly responsible for the degradation of proteins into smaller fragments or amino acids in senescent tissues, and its downregulation will result in enhancing plant senescence and pathogen-induced cell death (Singh et al., 2013). PCD-related genes are the fourth type of senescence-related-genes. For example, *RLS1* plays an important role in PCD regulation of plant senescence (Jiao et al., 2012), and *MADS29* regulates nucellar degradation and nucellar projection during plant seed development (Yin and Xue, 2012). Additionally, three transcription factor families, including NAC, TCP, and WRKY, have been characterized to be closely associated with senescence in several tissues, such as Arabidopsis leaves, petals, and siliques (Zhang et al., 2018; Wang et al., 2019a).

In addition to protein-coding RNAs, non-coding RNAs in gene regulation are also necessary for the growth and development of plants (Chekanova, 2015; Yu et al., 2019). MicroRNAs (miRNAs) are small, endogenous, non-coding RNAs with 19–24 nucleotides that regulate post-transcriptional events by splicing target mRNAs or blocking translation (Bartel, 2004). There is increasing evidence that miRNAs are involved in the regulation of seedlings growth, male and female infertility, flower development, and plant responses to senescence (Chen, 2004; Millar and Gubler, 2005; Yan et al., 2015; Hu et al., 2016). Some miRNAs are considered to be important participants in triggering plant senescence by regulating the expression level of their target genes. For example, miR164 and miR319 negatively regulate senescence-induced cell death by downregulating *ORE1* and *TCP* (Schommer et al., 2008; Kim et al., 2009). Long non-coding RNA (lncRNA) is a group of non-coding RNA longer than 200 nucleotides, which can regulate gene expression through cis/trans-acting or miRNA sponges (Liu et al., 2015a). Basically, lncRNAs play their roles through epigenetic modification, transcriptional regulation, and post-transcriptional regulation (Chen, 2016). With the rapid development of high-throughput sequencing technology, a large number of lncRNAs have been found in plant species, and it is considered that these lncRNAs might play crucial roles in a variety of biological processes (Liu et al., 2015b). However, only a handful of plant lncRNAs have been experimentally characterized, including *COOLAIR*, *APOLO*, *HID1*, *ELENA*, *IPS1*, etc. (Franco-Zorrilla et al., 2007; Ben Amor et al., 2009; Wang et al., 2014; Kim and Sung, 2017; Seo et al., 2017), among which two rice lncRNAs *PMS1T* and *LDMAR* have been shown to regulate photoperiod-sensitive male sterility (Ding et al., 2012; Zhou et al., 2012). In summary, despite of a growing number of documents on lncRNAs, their special functions in regulating plant senescence remain poorly understood. A competitive endogenous RNA (ceRNA) hypothesis proposes that lncRNAs, mRNAs, and pseudogenes can competitively bind to common miRNA response elements (MREs), thereby regulating various biological and developmental processes (Salmena et al., 2011; Meng et al., 2018; Yuan et al., 2018). In many studies, ceRNA regulation theory has been used to reveal the molecular mechanism of plant biology. For example, researchers have found that some lncRNAs (e.g., MSTRG.244.1 and MSTRG.16577.1) and the gene *OsSPL12* compete with osa-miR156 to form an lncRNA-miRNA-mRNA regulatory network that might be a potential ceRNA mechanism in response to glyphosate stress in rice (Zhai et al., 2020). Xu et al. (2016) have also constructed a ceRNA network to analyze the function of lncRNAs in rice adaptation to phosphate starvation. Moreover, ceRNA networks have been reported to be involved in tomato fruit development, corn anther development, and Arabidopsis plant development, respectively (Meng et al., 2018; Li et al., 2019; Yang et al., 2019). However, it is still now not clear how ceRNA networks regulate plant senescence.

As one of the world’s main food crops, rice feeds nearly half of the world’s population, but early senescence often leads to a decline in rice yield and quality (Lim et al., 2007). In the past decades, efforts to reveal the molecular mechanisms for rice early senescence were mainly made by pieces of transcriptomic (mRNA) and proteomic research (Kim et al., 2016). However, there have been few systematic reports that apply ceRNA networks to study coding and non-coding RNAs associated with rice senescence. In our previous work, a rice mutant library was constructed by mutagenesis of japonica rice (*Oryza sativa*) cultivar Zhejing22 (ZJ22) with ethyl methane sulfonate (EMS), and an early-senescence mutant, designated *zj-es* (*ZheJing-early senescence*), was characterized by screening the mutant library. Here, to elucidate the molecular regulation mechanism for early senescence in the mutant rice, the whole-transcriptome RNA sequencing was performed on *zj-es* and its wild type rice, and a large number of differentially expressed RNAs, including mRNA, miRNA, and lncRNAs, were identified and extensively analyzed. In addition, the related phenotypic and physiological analysis was conducted on the both rice materials. Based on the data and analysis above, we suggest that ROS-induced different autophagosomes play a key role in the PCD and its coupled early senescence in *zj-es* mutant rice. These results of this study will make great contribution to full exploration for the regulation mechanism of early senescence in rice.

## Results

### Phenotypic Characterization of *zj-es*

**FIGURE 1:**
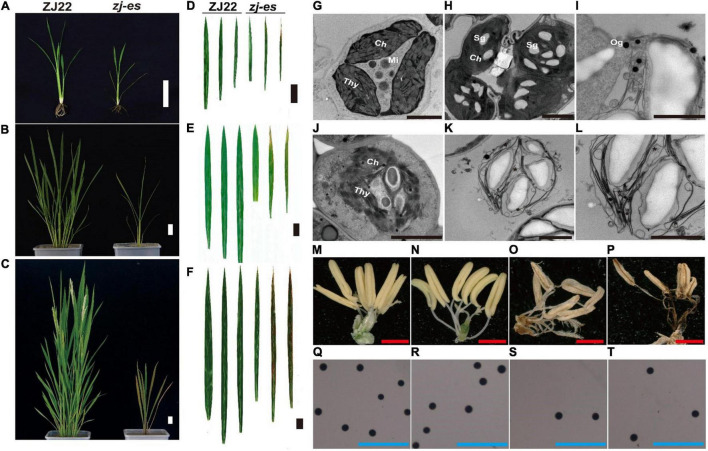
Comparison of plant morphology between the wild type (ZJ22) and mutant (*zj-es*). The whole plant at the six-leaf stage **(A)**, the tillering stage **(B)**, and the adult stage **(C)**, scale bar = 5 cm; leaf genotype at the six-leaf stage **(D)**, the tillering stage **(E)**, and the adult stage **(F)**, the three leaves represent the flag leaf, 2nd leaf (from the top), and 3rd leaf; scale bar = 3 cm; ultrastructure of the chloroplast in the ZJ22 **(G)** and *zj-es* **(H–L)** at the six-leaf stage; Sg, starch grain; Og, osmiophilic granules; Ch, chloroplast; Mi, mitochondria; Thy, thylakoid, scale bar = 0.5 nm; anther of ZJ22 **(M,N)** and of *zj-es* **(O,P)**, red scale bar = 1 mm; pollen grains of ZJ22 **(Q,R)** and of *zj-es* **(S,T)** after potassium iodide staining, blue scale bar = 500 μm.

Similar to the simulated early senescence, *zj-es* mutant showed senescence from the seedling stage to the adult-plant stage. In *zj-es*, at the six-leaf stage, a large number of reddish-brown lesions occurred on the third leaf, smaller numbers on the second leaf, and normal green appearance on the flag leaves. The tip of the spotted leaves began to chlorosis, and the area of spots was mainly concentrated near the tip of the leaf (Figures 1A,B). At the tillering stage, the symptoms of early senescence were aggravated. A large number of reddish-brown spots were appeared in the second and third leaves of *zj-es*, and the area of the spots spread to the middle and below the leaves, while the flag leaves almost had no spots (Figures 1C,D). At the adult stage, the symptoms of early senescence were the most serious, with reddish-brown lesions in all leaves of the whole plant. The spots were distributed in the whole leaves, and the chlorosis area of leaves increased (Figures 1E,F). Transmission Electron Microscope (TEM) analysis showed that the starch grains and osmium globules in the spotted leaves of *zj-es* were larger and more numerous than those in its wild type rice ZJ22 (Figures 1G–L), which is more consistent with the characteristics of the early stages of leaf senescence (Zhao et al., 2018). Furthermore, compared with ZJ22, the chloroplast structure became loose and the lamellar structure began to collapse in *zj-es*. Even in some chloroplasts, the thylakoid membrane disintegrated, the layered structure disappeared, and membrane-like residues appeared in mesophyll cells (Figures 1J–L), which is also consistent with the reported characteristics of early senescence cells (Hong et al., 2018). In addition, the anthers of the two kinds of rice were compared morphologically (Figures 1M–T). The anthers of ZJ22 were yellow and full, while those of *zj-es* were light yellow and atrophic in the early stage of maturity, and gradually appeared brown and shriveled in the late stage (Figures 1O,P). Moreover, the pollen grains of ZJ22 were numerous, large, and full after potassium iodide staining, and, in comparison, *zj-es* exhibited much less pollen grains (Figures 1S,T), which indicated that the fertility of *zj-es* was declined. We also detected the main agronomic traits in both rice materials, and the results showed that plant height, dry weight, wet weight, effective panicle number, grain number per panicle, and 1,000-grain weight were all significantly decreased in *zj-es* (Table 1).

**TABLE 1:** Comparison of agronomic traits between ZJ22 and *zj-es*.

Trait		ZJ22	*zj-es*
Plant height (cm)	Six-leaf stage	27.02 ± 1.46	16.25 ± 2.01**
	Tillering stage	61.22 ± 2.06	46.98 ± 1.48**
	Adult stage	111.66 ± 3.63	56.52 ± 8.36**
Fresh weight (g)	Six-leaf stage	2.73 ± 0.16	1.67 ± 0.18*
	Tillering stage	41.16 ± 5.41	3.96 ± 0.51**
	Adult stage	228.51 ± 56.13	9.95 ± 2.27**
Dry weight (g)	Six-leaf stage	0.17 ± 0.02	0.09 ± 0.03*
	Tillering stage	7.28 ± 0.95	0.63 ± 0.07**
	Adult stage	59.58 ± 18.12	1.60 ± 0.37**
Tiller number		14.33 ± 1.53	1.67 ± 0.58**
Growth duration		132.33 ± 2.52	146.67 ± 4.16**
Number of productive panicles per plant		13.33 ± 0.58	0.33 ± 0.58**
Grain number per panicle		155.79 ± 24.84	1.33 ± 1.15**
1000-grain weight (g)		25.66 ± 0.79	5.53 ± 0.17**

Mean ± SD, n = 10, *significance at p < 0.05, **extremely significant at p < 0.01.

### Mapping of the Gene Locus for *zj-es*

**FIGURE 2:**
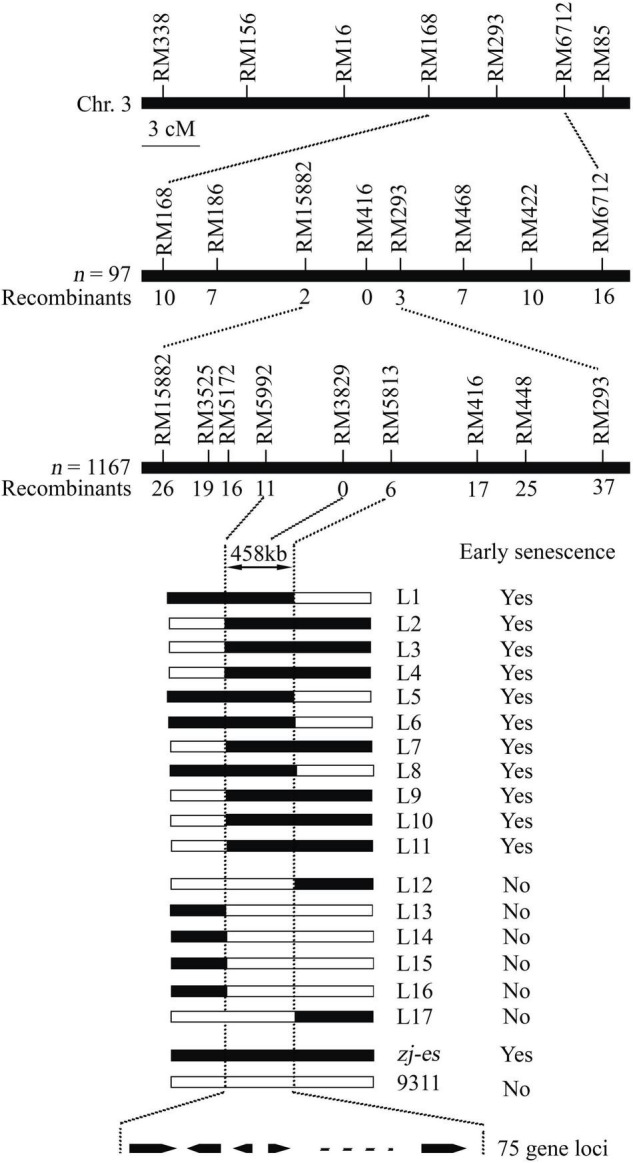
Molecular mapping of the *ZJ-ES* gene on Chromosome 3. *ZJ-ES* locus was initially mapped in a region between markers RM15882 and RM293 on Chromosome 3 and was further narrowed to about 458 kb region between markers RM5992 and RM5813 with 75 predicted gene loci. The numbers of individuals in F2 population and the recombinants of each marker are indicated.

Whether *zj-es* (japonica) was crossed with ZJ22 (japonica) or with another rice variety 9311 (indica), the phenotype of all individual F1 plants was normal. In F2 population of the both cross types, individual plants showed the segregation of wild phenotype and early senescence phenotype in a ratio of about 3:1 (χ^2^ = 1.24 < χ^2^_0_._05_ = 3.84, *zj-es*/ZJ22; χ^2^ = 0.56 < χ^2^_0_._05_ = 3.84, *zj-es*/9311, *p* > 0.05) (Supplementary Table 1). Genetic analysis above indicated that the early senescence phenotype of *zj-es* was controlled by a single recessive nuclear gene. The *ZJ-ES* gene was initially mapped in a region between molecular markers RM168 and RM6712 on Chromosome 3 using an F2 population of *zj-es*/9311, including 97 individuals (Figure 2). Subsequently, we enlarged the F2 population with 1,167 individuals, through which we narrowed the mapping region to a smaller chromosome interval between markers RM15882 and RM293. According to the rice genome annotation project website,^1^ the physical distance of the target region is 458 kb between markers RM15992 and RM5813, and contains 75 predicted gene loci (Supplementary Table 2). None of the 75 predicted genes are same as these reported genes controlling early senescence phenotype of rice (Supplementary Table 2), which suggests that the *ZJ-ES* gene is a novel gene of rice early senescence.

### Sequencing Quality Control and Mapping Results

According to the unique leaf phenotype of the *zj-es* mutant, leaves from three different leaf positions were collected for RNA sequencing: the mutant’s flag leaf was designated as *zj-es* a, the second leaf (from the top) as *zj-es* b, and the third leaf as *zj-es* c, while the wildtype flag leaf as ZJ22a, the second leaf as ZJ22b, and the third leaf as ZJ22c, and each line comprises three biological duplications.

RNA was extracted individually from 18 samples of *zj-es* and ZJ22, and double-stranded cDNA was synthesized. After sequencing, fastp (Chen et al., 2018) was used to perform quality control on the raw reads that were offline. Based on the raw data, clean reads were obtained by removing the data containing adapters and Poly A base, and removing low-quality data (the number of bases with Q ≤ 20 accounts for more than 50% of the entire read). Finally, a total of 1,473,241,334 raw reads were generated from cDNA libraries. Approximately, 94% of clean reads were mapped to the reference genome, among which nearly 44% were mapped to unique locations (Supplementary Table 4). These results provide massive data for further analyses on expression profiling and the metabolism pathway of mRNAs and lncRNAs.

### Identification of Differentially Expressed mRNAs, lncRNAs, and miRNAs

#### Identification of Differentially Expressed mRNAs

The consistency between samples was tested by calculating Pearson’s correlation coefficient (*R* value) between every two samples. The minimum *R* value for comparison in the three biological replicates of each sample was 99.02%, most of which were 99.35–99.98%. Principal component analysis (PCA) was performed with R package g models^2^ in this experience. PCA analysis also showed close correlation between repeated samples, which proved that the repeatability of the samples and subsequent analysis could be continued (Supplementary Figure 1).

**FIGURE 3:**
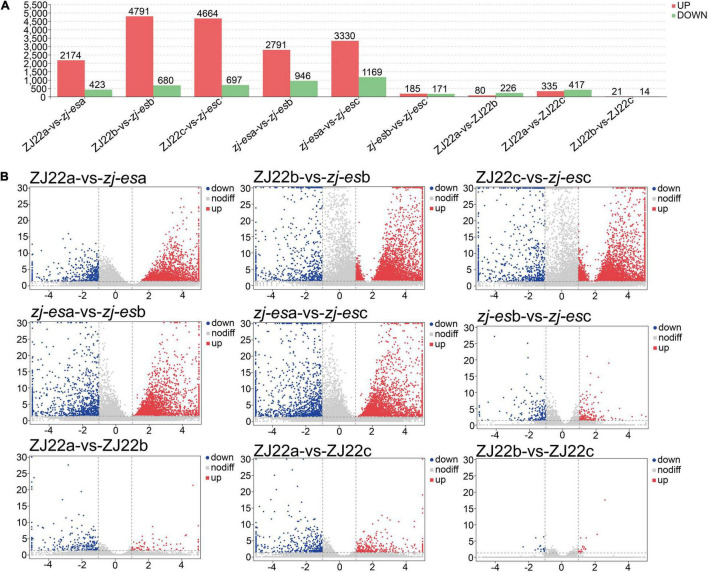
Analysis of differentially expressed mRNAs in each comparison group. **(A)** The number of significantly regulated genes. **(B)** Volcano plots of mRNAs expression levels.

Considering the parameter of FDR (false discovery rate) under 0.05 and absolute fold change ≥ 2 as differentially expressed mRNAs (DEmRNAs), a total of 23,112 DEmRNAs were identified between *zj-es* and ZJ22, among which 18,370 genes were significantly upregulated and 4,742 were downregulated (Figure 3A and Supplementary Table 5). Intuitively, among the DEmRNAs of all comparison groups, the difference trend was that the number of upregulated mRNAs was much greater than that of downregulated genes. The volcano map (Figure 3B) showed the gene expression profiles of the DEmRNAs in nine comparison groups. The up/downregulated genes had a clear distribution pattern, which showed that a distinct expression pattern existed in different comparison groups. The difference between up and downregulation mRNAs was much more significant in the four groups of ZJ22b vs. *zj-es*b, ZJ22c vs. *zj-es*c, *zj-es*a vs. *zj-es*b, and *zj-es*a vs. *zj-es*c than in the other ones, which indicated that the differences between leaves with and without early senescence and mild and severe early senescence were the largest.

#### Identification of Differentially Expressed lncRNAs

**FIGURE 4:**
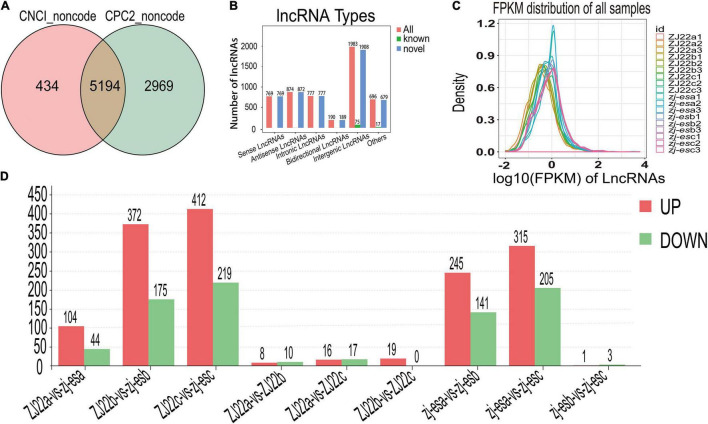
Identification of differentially expressed lncRNAs (DElncRNAs). **(A)** Venn diagrams of coding potential of lncRNAs. **(B)** Types of lncRNAs. **(C)** An abundance distribution map of lncRNAs. Each color represents a sample. **(D)** Number of significantly regulated genes of lncRNAs.

To obtain high-quality data, two kinds of software CNCI (version 2) (Sun et al., 2013) and CPC (version.9-r2) (Kong et al., 2007)^3^ were used to assess the protein-coding potential of novel transcripts, through which 8,597 transcripts from all the 19,174 identified transcripts were predicted to be possible lncRNAs. The intersection of non-protein-coding transcripts predicted by both kinds of software was chosen as the candidate lncRNAs for further analysis, and, at last, a total of 5,289 lncRNAs from ZJ22 and *zj-es* were identified (Figure 4A). After screening known rice lncRNAs in the GREENC database, we identified 95 known lncRNAs and 5,194 novel lncRNAs in *zj-es* and ZJ22 (Supplementary Table 6). LncRNAs were classified into five types according to their location relative to protein-coding genes: intergenic lncRNAs, bidirectional lncRNAs, intronic lncRNAs, antisense lncRNAs, and sense overlapping lncRNAs. Among the five types of lncRNAs, the number of intergenic lncRNAs was the largest (1,908 novel lncRNAs, 75 known ones), and that of bidirectional lncRNAs was the least (189 novel lncRNAs, 1 known ones) (Figure 4B).

For each transcription region, an FPKM (fragment per kilobase of transcript per million mapped reads) value can be calculated to quantify its expression abundance and variations using the StringTie software. The FPKM distribution of the 18 samples reveals their fundamental expression pattern. As shown in Figure 4C, the curve of density-FPKM exhibited the same trend in all of the samples, which indicated that lncRNAs expression levels could be totally comparable among all the groups with reliable repeatability.

At a cutoff with an absolute value of log_2_FC > 1 and FDR < 0.05, we identified 2,306 differentially expressed lncRNAs (DElncRNAs) between *zj-es* and ZJ22, among which 64.7% genes were significantly upregulated and 35.3% were downregulated (Figure 4D). The total number of significant regulatory genes differed in different comparisons. The number of downregulated genes was greater than that of upregulated genes in three comparison groups (ZJ22a vs. ZJ22b, ZJ22a vs. ZJ22c, and *zj-es*b vs. *zj-es*c), while the other comparison groups had the opposite trend. Interestingly, the differences of lncRNAs regulation were much more striking in the comparison groups of ZJ22b vs. *zj-es*b, ZJ22c vs. *zj-es*c, *zj-es*a vs. *zj-es*b, and *zj-es*a vs. *zj-es*c than in other ones, which indicated that, to some extent, these DElncRNAs might be involved in triggering and aggravating the process of rice early senescence.

#### Identification of Differentially Expressed miRNAs

Apart from mRNAs and lncRNAs, a total of 495 existed miRNAs, 555 known miRNAs, and 561 novel miRNAs were identified using Bowtie (version 1.1.2) software (Supplementary Table 7). The identified miRNAs were summarized in each sample, and then the TPM (tags per million) expression of each miRNA was calculated. Finally, the expression profile of all miRNAs was obtained in all samples. The differential expression analysis of miRNAs was performed by the edgeR software between different groups. We identified miRNAs with a fold change ≥ 2 and *P*-value < 0.07 in a comparison as significant differentially expressed miRNAs (DEmiRNAs). Totally, 88, 218, 197, 135, 260, 68, 174, 268, and 90 DEmiRNAs were identified between ZJ22a and *zj-es*a, ZJ22b and *zj-es*b, ZJ22c and *zj-es*c, ZJ22a and ZJ22b, ZJ22a and ZJ22c, ZJ22b and ZJ22c, *zj-es*a and *zj-es*b, *zj-es*a and *zj-es*c, *zj-es*b and *zj-es*c, respectively (Supplementary Figure 2). The largest DEmiRNAs number was found between *zj-es*a and *zj-es*c (268), followed by ZJ22a and ZJ22c (260), and then ZJ22b and *zj-es*b (218). In the *zj-es*a vs. *zj-es*c comparison group (180 upregulated miRNAs, 88 downregulated ones), the difference adjustment ratio was largest, and this ratio was smallest in the ZJ22b vs. ZJ22c group with equal number of up and downregulation miRNAs. Based on these data, scatter plot analysis was performed to visually display the miRNA differences between the comparison groups, and the results showed that the expression patterns were clear between different groups (Supplementary Figure 3).

#### Gene Ontology/Kyoto Encyclopedia of Genes and Genomes Pathways Analysis of Differentially Expressed mRNAs and miRNAs

To explore the potential function of DEmRNAs, Gene Ontology (GO) and Kyoto Encyclopedia of Genes and Genomes (KEGG) analyses were performed. Based on GO analysis, we found that most DEmRNAs in *zj-es* and ZJ22 were annotated to the cellular process, metabolic process, single-organism process, and biological regulation under the biological process (BP) to cell, cell part, organelle, and membrane under cellular component (CC), and to binding, catalytic activity, transporter activity, and structural molecule activity under molecular function (MF) (Supplementary Figure 4 and Supplementary Table 8). In these enriched GO terms, the number of upregulated DEmRNAs was far more than that of downregulated ones. The number and classification of DEmRNAs annotated by GO analysis had similar distribution patterns in different comparison groups. The DEmRNAs were enriched in KEGG pathways, and the obviously enriched KEGG pathways are shown in Supplementary Figure 5 and Supplementary Table 9. In the nine comparison groups, DEmRNAs were mainly enriched in metabolic pathways, biosynthesis of secondary metabolites, peroxisome, fatty acid degradation, and valine, leucine, and isoleucine degradation (Supplementary Figure 5).

We also conducted GO and KEGG analyses for the target mRNAs of miRNAs. The results showed that all target mRNAs were successfully annotated into the corresponding 26 GO terms. The main subcategories were the metabolic process, cellular process, cell part, binding and catalytic activity (Supplementary Table 10). The target mRNAs were enriched in 32 KEGG pathways (Supplementary Figure 6 and Supplementary Table 11), among which significantly in six ones, including metabolic pathways, endocytosis, starch and sucrose metabolism, lysine degradation, and ubiquinone and other terpenoid-quinone biosynthesis. *P*-values greater than 0.05 for other pathways indicated no statistically significant difference and, therefore, no explanation.

### Identification of Weighted Gene Co-expression Network Analysis Modules Associated With Early Senescence

**FIGURE 5:**
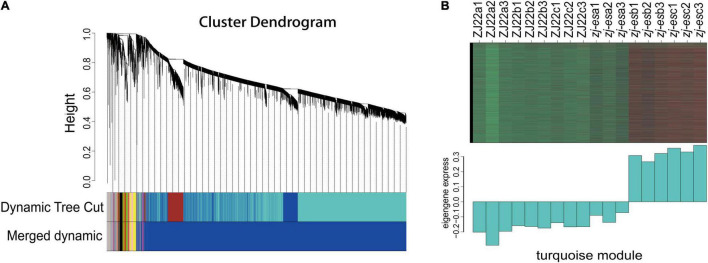
WGCNA of genes identified in ZJ22 and *zj-es*. **(A)** Eight modules of co-expressed genes are shown in a hierarchical cluster tree. **(B)** The expression profile of all the co-expressed genes in the turquoise module. The upper panel is the heat map of gene expression levels in different samples (red, upregulation; green, downregulation); the lower panel is the consensus expression pattern of the corresponding co-expressed genes in this module.

To more comprehensively analyze the association between early senescence phenotype and *ZJ-ES* gene regulation, we performed a weighted gene co-expression network analysis (WGCNA). A gene cluster scheme with a power value of 7 was constructed, and the filtered genes with similar expression patterns were divided into the 8 modules (Figure 5A). By associating the gene expression profile of each module with all samples, a heat map of module-sample matrix was generated (Supplementary Figure 7), and we found that the gene expression profiles in the turquoise module were closely consistent with the phenotype of early senescence (Figure 5B), which indicated that the genes in this module might trigger and participate the process of rice early senescence.

**FIGURE 6:**
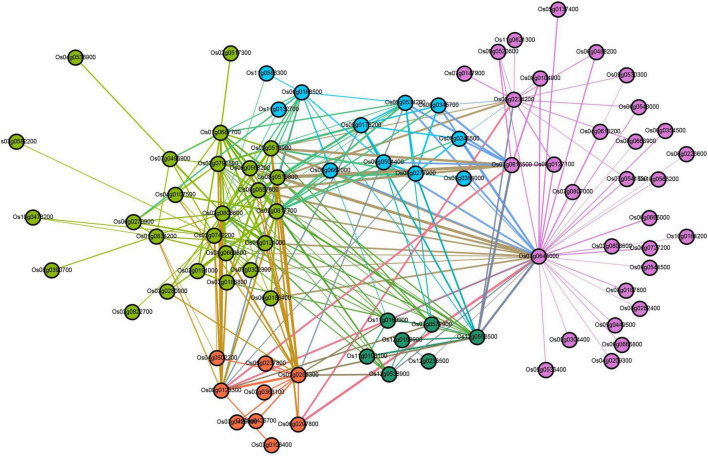
Co-expression regulatory network analysis of turquoise module.

In the turquoise module, there are a total of 6,859 differentially expressed genes (DEGs) involved in cell phagocytosis, metabolic processes, synthetic processes, and energy. GO annotation of the unigenes showed that they were annotated in 22 terms under BP (mainly involved in the cellular process, metabolic process, single-organism process, and biological regulation), 17 terms under CC (mainly involved in cells, cell part, organelle, membrane), 14 terms under MF (mainly involved in binding, catalytic activity, transporter activity, and nucleic acid binding transcription factor activity) (Supplementary Table 12). KEGG analysis showed that DEGs in turquoise modules were significantly enriched in autophagy-other eukaryotes, peroxisome, phagosome, SNARE interactions in vesicular transport, and endocytosis (Supplementary Table 13). In addition, genes in these pathways as potential hub genes to construct a gene regulatory network (Figure 6). This network analysis results showed that there is complex (direct and indirect) cross-regulation between these central genes, for example, beclin-1 (Os03g0644000) and ras-related protein (Os06g0234200) were the core genes in purple nodes; calmodulin-binding protein (Os12g0556500) and OsSub49-Putative subtilisin homolog (Os06g0163500) are the core genes of the green and blue nodes, respectively, which were closely related to phagocytosis and were highly expressed in the second and third leaves of *zj-es*. Accordingly, we speculated that these DEGs were associated with the senescence process of the second and third leaves of *zj-es*. And the regulation of them might lead to plant senescence through the production of autophagosomes.

Furthermore, DEGs were used as the target genes to find upstream transcription factors (TFs), and we established a co-expression regulatory network by combining TFs with the genes that were predicted to be regulated by these TFs (Supplementary Figure 8). The results showed that 31 TF genes (red nodes in Supplementary Figure 8) from two families, including FAR1 and LFY, were considered to be the top 31 highly connected TFs (hub TFs) in this regulatory network (Supplementary Figure 8 and Supplementary Table 14). The purple nodes represent the predicted regulatory genes of these key TFs. In general, TFs and their predicted regulatory genes showed a many-to-many relationship, with multiple TFs regulating any one predicted gene and individual TFs regulating multiple predicted genes.

### Construction and Analysis of ceRNA Network of lncRNA-miRNA-mRNA

**FIGURE 7:**
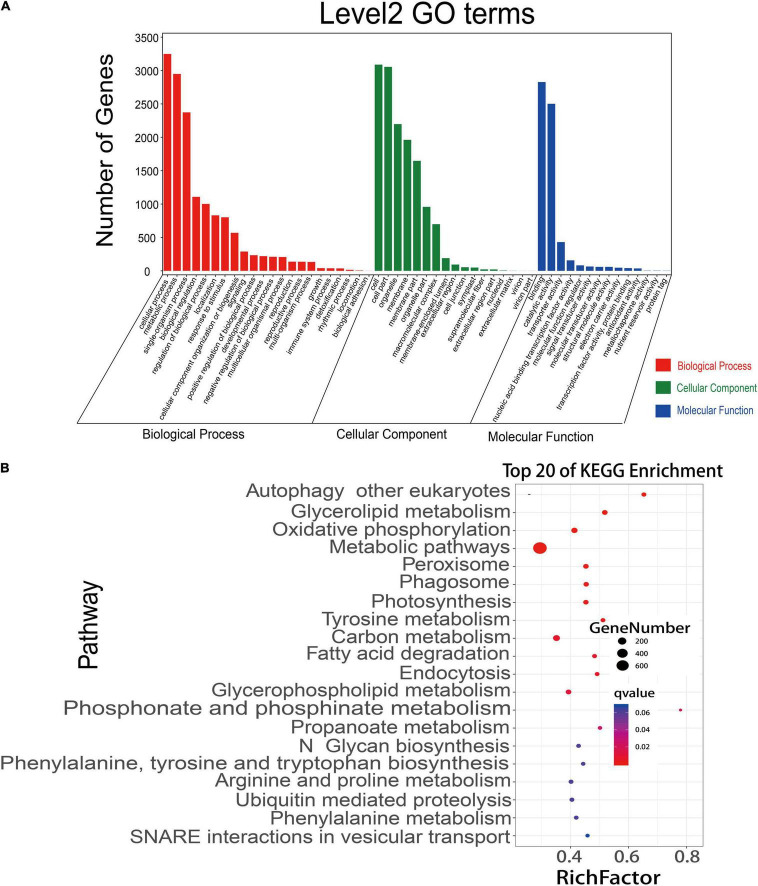
GO annotations and KEGG pathway analyses of differentially expressed mRNAs. **(A)** mRNAs were significantly enriched in GO terms (*P*-value < 0.05). **(B)** KEGG pathways involving the top 20 terms. *Q*-value ranges from 0 to 1. The closer to zero the *Q*-value is, the more significant the enrichment is. A larger RichFactor value indicates a higher degree of enrichment.

A competitive endogenous RNA (ceRNA) regulatory network was constructed using the data of DEmiRNAs, DEmRNAs, and DElncRNAs. A total of 12,739 matched lncRNA-mRNA pairs were identified from 9,214 DEmRNAs and 4,569 DElncRNAs. Meanwhile, 44,700 matched miRNA-mRNA pairs were found from 20,904 DEmRNAs and 1,611 DEmiRNAs (Supplementary Table 15). To more accurately analyze the role of these different RNAs in the process of rice early senescence, we intersected these matched pairs mRNAs-miRNAs and mRNAs-lncRNAs with the genes in the turquoise module of WGCNA. Ultimately, a total of 190 mRNA-lncRNA pairs and 3,391 mRNA-miRNA pairs were obtained (Supplementary Table 15). To reveal their potential function, we performed GO and KEGG pathway analyses of DEmRNAs to predict the potential regulatory role of lncRNAs in the ceRNA network. The results showed that these mRNAs were involved in the regulation of many biological processes, including cellular processes, response to stimulus, and the metabolic process (Figure 7A). In the KEGG analysis, the genes were involved in 35 different pathways, and, among the top pathways, there were four ones related to the autophagy pathway, including autophagy, phagosome, endocytosis, and vesicular transport (Figure 7B). Additionally, another pathway of peroxisome produces ROS that may play an important role in the autophagy pathway (Figure 7B). Up to seven pathways were associated with the metabolism of molecular substances, including glycerolipid metabolism, tyrosine metabolism, fatty acid degradation, glycerophospholipid metabolism, arginine and proline metabolism, phenylalanine metabolism, and ubiquitin-mediated proteolysis (Figure 7B), while molecular metabolism is one of the most important events in the autophagy pathway. Therefore, the results here strongly suggested that autophagy might involve in the early senescence of *zj-es*.

**FIGURE 8:**
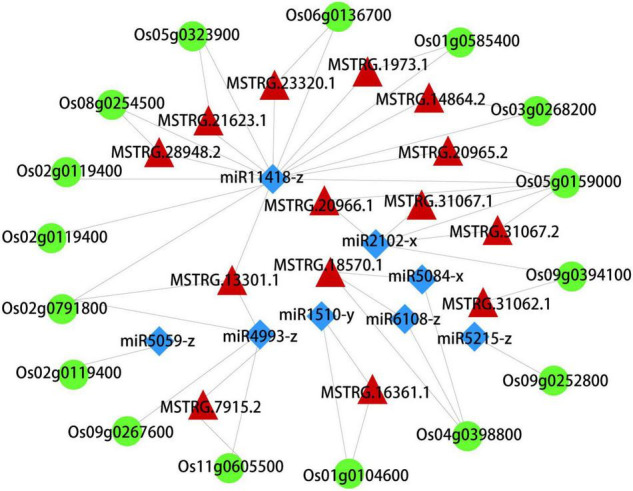
A CeRNA network constructed with DEmRNAs, DElncRNAs, and DEmiRNAs. Green, red, and blue nodes represent mRNA, lncRNA, and miRNA, respectively.

What interests us is the potential regulation mechanism of these autophagosomes metabolism in the early senescence of *zj-es*, so we constructed the ceRNA network using Cytoscape_3.3.0 software based on the data of mRNAs, lncRNAs, and miRNAs from the six metabolic pathways of peroxisome, autophagy, phagosome, endocytosis, vesicular transport, and ubiquitin-mediated proteolysis. This network comprised 16 mRNAs, 8 miRNAs, and 14 lncRNAs, and there existed intricate relationships among these ceRNAs as shown in Figure 8. For example, Os03g0268200, Os02g0791800, Os06g0136700, and Os01g0585400 were predicted to be the target genes for miR11418-z. Os04g0398800 was predicted to be the target gene for miR5084-x and miR6108-z. Os02g0791800, Os09g0267600, and Os11g0605500 were predicted to be the target genes for miR4993-z. Meanwhile, the lncRNAs MSTRG.14864.2, MSTRG.13301.1, MSTRG.21623.1, MSTRG.28948.2, MSTRG.20965.2, MSTRG.1973.1, and MSTRG.23320.1 were predicted to bind to miR11418-z. MSTRG.31062.1, MSTRG.31067.1, MSTRG.31067.2, and MSTRG.20966.1 were predicted to bind to miR2102-x. Os03g0268200 (serine/threonine-protein kinase), Os02g0791800 (WD-40 repeat), and Os08g0254500 (chloroplast precursor SecY) were the vital genes of autophagy and phagosome. Os06g0136700 (steroid nuclear receptor), Os04g0398800 (H0209H04.5 protein), and Os09g0267600 (charged multivesicular body protein 4b) were the vital genes of endocytosis. In short, this complex ceRNA network indicated the regulation mechanism of the autophagosomes metabolism in triggering the early senescence of *zj-es.*

### Validation of DEmRNAs, DElncRNAs, and DEmiRNAs by qRT-PCR

**FIGURE 9:**
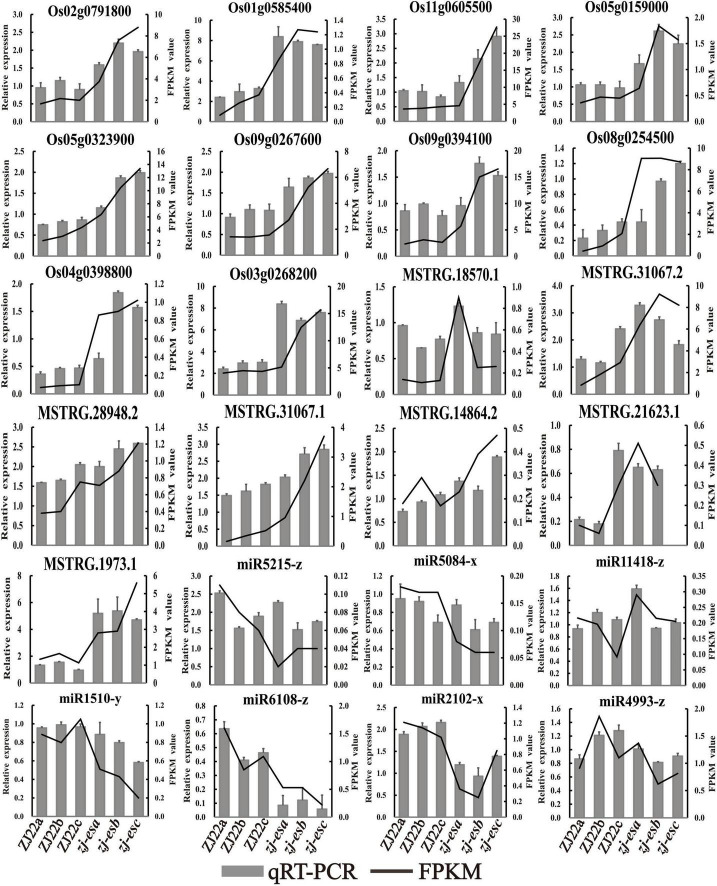
The expression profiles of qRT-PCR were compared with the results of RNA-Seq analysis. The black line means FPKM, and the gray column means qRT-PCR.

To ensure the quality of high-throughput sequencing, several mRNAs, lncRNAs, and miRNAs were selected for qRT-PCR detection. The validated results are shown in Figure 9, and the primers are listed in Supplementary Table 16. In terms of the expression trend, qRT-PCR results were consistent with RNA-seq data. However, validation results of ncRNAs may differ from the sequencing data due to the different principles of detection methods.

### Subcellular Localization Analysis of Selected Genes

**FIGURE 10:**
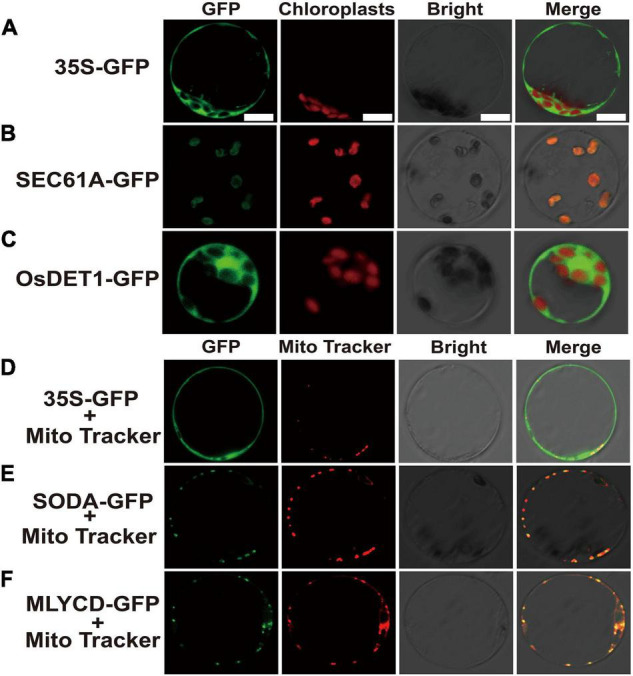
Confocal microscope observation of subcellular localization with green fluorescent protein (GFP) fusion organelle markers in rice protoplasts. Subcellular localization of negative control **(A)**, SEC61A **(B)** and OsDET1 **(C)**. Negative control **(D)**, SODA-GFP **(E)** and MLYCD-GFP **(F)** co-localized with mitochondria tracker. GFP, Take photographs under the 390-nm excitation light, Bright: Take photographs under the bright light. Scale bar = 10 μm.

After the genes were fused with green fluorescent protein (GFP), the subcellular localization in rice protoplasts could clearly exhibit the position of the target genes in the cell, which is helpful for further understanding the possible mechanism of the related genes in regulating early senescence of *zj-es*. Subcellular localization vectors of four DEGs, *SEC61A* (Os08g0254500), *OsDET1* (Os01g0104600), *SODA* (Os05g0323900), and *MLYCD* (Os09g0394100) were constructed to transform rice protoplasts. Under confocal laser scanning microscopy, it was found that the protein OsDET1 was located in the nucleus and pericellular, SEC61A in the chloroplast, and SODA and MLYCD in the mitochondria (Figure 10). These results also indicate that multiple cellular components of the cell may be involved in the triggering of the early senescence mechanism synergistically.

### Autophagosomes and Cell Death Occurred in *zj-es*

**FIGURE 11:**
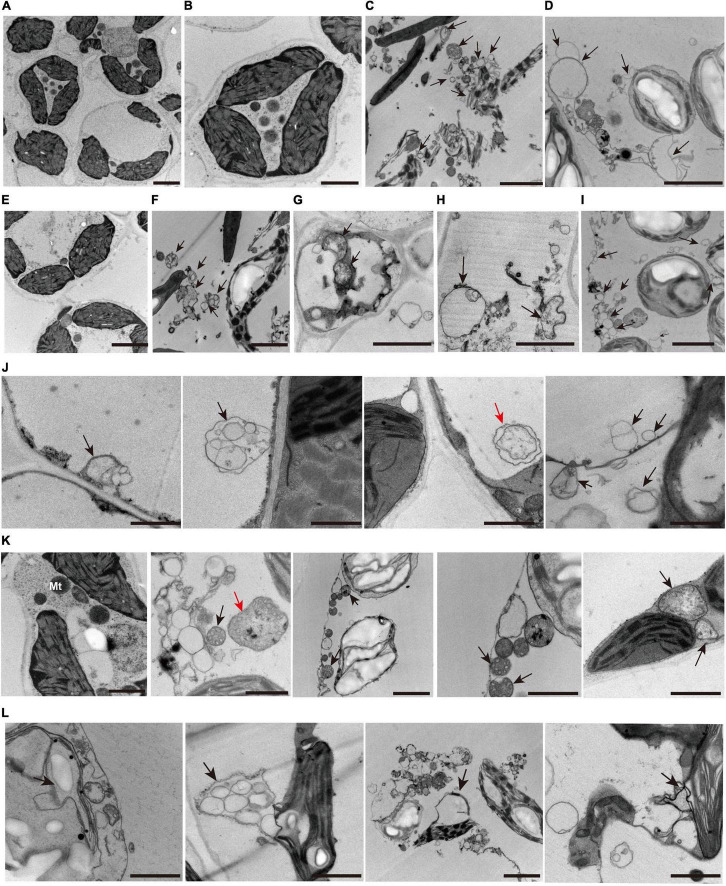
Representative transmission electron microscope (TEM) images of autophagosome structures. **(A–I)** Representative ultrastructure of autophagosomes (arrows) observed of mesophyll cells of wild-type ZJ22 **(A,B,E)** and mutants *zj-es* **(C,D,F–I)**. **(J)** The plasma membrane was involved in the formation of autophagosomes (arrows). **(K)** The mitochondria were involved in the formation of autophagosomes (arrows). **(L)** The chloroplast membrane was involved in the formation of autophagosomes (arrows). The red arrows show the classic double-membrane autophagosomes.

Analysis from whole-transcriptome RNA sequencing revealed that many metabolic pathways that are related to autophagy might play a key role in the regulation of *zj-es* early senescence (Figure 7). A large number of autophagosomes were also found in mesophyll cells of *zj-es* mutant through TEM images (Figure 11). These autophagosomes exhibited a variety of types, with different sizes and shapes (Figures 11F–I). The membranes of these autophagosomes were found to tightly link to plasma membranes or subcellular organelles, such as chloroplasts and mitochondria (Figures 11J–L), which indicates that their membrane structure might be from the degraded organelles. These observations suggested that autophagosomes were involved in the occurrence of early senescence of *zj-es*, which was consistent with the results of whole-transcriptomes.

**FIGURE 12:**
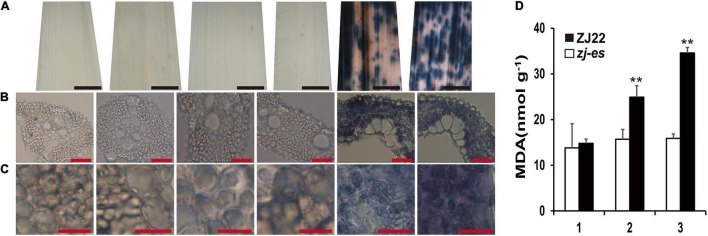
Trypan blue (TB) staining and the determination on the leaves of ZJ22 and *zj-es* sampled at the six-leaf stage. Each set of images represents (from left to right) the flag, second and third leaves of ZJ22, and then the flag, second and third leaves of *zj-es*. **(A)** Leaf phenotype; red scale bar = 0.2 cm. **(B)** Cell morphology; scale bar = 50 μm. **(C)** Magnified cell morphology, scale bar = 10 μm. **(D)** The content of malonaldehyde (MDA) in the wild-type ZJ22 and *zj-es* mutant at the six-leaf stage. 1: flag leaves; 2: second leaves from top; 3: third leaves from top. **Significant difference at *p* < 0.01.

Trypan blue (TB) can detect cell death and thus prove the production of PCD. As shown in Figure 12, numerous dark blue spots appeared in the second leaf of *zj-es*, and the whole third leaf was almost covered with blue spots, whereas all of the other samples only showed light blue staining, including the flag leaf of *zj-es* and the leaves from three-leaf positions of ZJ22 (Figure 12A). Under the microscope, it was also found that the mesophyll cells from the leaves of *zj-es* were stained much more obviously than those from ZJ22 (Figures 12B,C). All results here suggested that massive cell death occurred in the leaves of *zj-es*. Malonaldehyde (MDA) content can reflect the degree of lipid peroxidation, which indirectly reflects the degree of cellular damage (Li et al., 2014). We measured MDA content at the six-leaf stage (Figure 12D). As expected, compared to ZJ22, the *zj-es* mutant showed significantly increased MDA in leaves, which could be PCD-related cellular damage.

### Reactive Oxygen Species Production System

**FIGURE 13:**
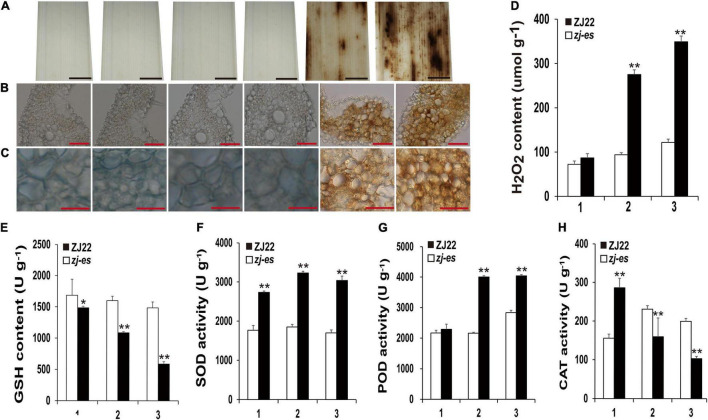
Diaminobenzidine (DAB) staining and the determination of enzyme activities involved in scavenging and generating the mechanism of ROS. **(A–C)** DAB staining for ROS accumulation of ZJ22 and *zj-es* mutant leaves (From left to right, each image representing the flag, second and third leaves of ZJ22, and then the flag, second and third of *zj-es*). **(D)** The content of hydrogen peroxide (H_2_O_2_) in the wild-type ZJ22 and *zj-es* mutant at the six-leaf stage. **(E–H)** The enzyme activities of glutathione (GSH), superoxide dismutase (SOD), peroxidase (POD), and catalase (CAT) in the wild-type ZJ22 and *zj-es* mutant at the six-leaf stage. 1: flag leaves; 2: second leaves from top; 3: 3rd third leaves from top. *Significant difference at *p* < 0.05; **Significant difference at *p* < 0.01.

Many reports have revealed that ROS as a signal molecule can induce the generation of autophagosomes (Cuervo, 2004; Xiong et al., 2005; Yu et al., 2020), and our work also showed that some DEGs were enriched in the pathway of peroxisome, so we detected H_2_O_2_ and different redox enzymes in both ZJ22 and *zj-es* to study the possible role of ROS in autophagy and rice early senescence. Diaminobenzidine (DAB) staining showed that numerous reddish-brown spots were found in the leaves of *zj-es*, especially in second and third leaves (Figure 13A), and, under the microscope, the relative mesophyll cells were also stained bright reddish-brown (Figures 13B,C), which indicated that H_2_O_2_ was largely accumulated in the leaves of *zj-es*. We also directly determined H_2_O_2_ content in the leaves of *zj-es* and its wild rice. H_2_O_2_ content in the flag leaf hardly exhibited a significant difference between both rice materials, while the content in second and third leaf was much higher in *zj-es* than in ZJ22, respectively (Figure 13D). The decrease of glutathione (GSH) content is a potential early activation signal of senescence, which can induce the production of oxygen free radicals, and then produce H_2_O_2_ (Winterbourn and Metodiewa, 1994; Jones, 2006). As expected, GSH content was significantly decreased in leaves of *zj-es* in comparison with ZJ22 (Figure 13E). In plants, superoxide dismutase (SOD) catalyzes the disproportionation of superoxide anion radical to produce H_2_O_2_ and oxygen, and peroxidase (POD) can also oxidize some substances with H_2_O_2_ to produce other types of ROS. Compared with ZJ22, *zj-es* demonstrated a notably increased tendency in the activity of SOD and POD (Figures 13F,G), which meant that ROS were largely generated in the leaves of mutant rice. In the second or third leaf, catalase (CAT) activity was much lower in *zj-es* than in ZJ22 (Figure 13H). CAT is one of the important antioxidant enzymes in plants, which catalyzes the decomposition of H_2_O_2_ into water and oxygen, so the remarkable decrease in CAT activity might cause the accumulation of H_2_O_2_ in the senescent leaves of mutant rice. Taken together, results from both H_2_O_2_ detection and analysis of oxidoreductase activity showed that a large number of ROS were produced and accumulated in mutant rice.

## Discussion

### *ZJ-ES* Gene Is a Novel Gene for Rice Early Senescence

Rice early senescence is a complex process, and its detailed mechanism still remains poorly understood. In this study, the mutant rice *zj-es* exhibited typical early-senescence features, including leaf lesions, chloroplast disintegration, plant dwarf, reduced fertility, and decreased agronomic traits (Figure 1 and Table 1), which suggests that this mutant is an important material for studying the regulation mechanism of rice early senescence.

Genetic analysis indicated that the early senescence phenotype of *zj-es* was controlled by a single gene (Supplementary Table 1), and the target *ZJ-ES* gene was mapped to the 458 kb chromosome region that contained 75 predicted gene loci (Figure 2). We attempted to further identify the possible candidate gene for *ZJ-ES* by analyzing the sequence mutations and differential expression of 75 predicted genes. However, there were no SNPs or Indels in these sequences (Supplementary Table 3), suggesting that *ZJ-ES*-related mutation may be located in the intergenic region or promoter sequence. Differential expression analysis of these predicted genes cannot also help us discover the candidate *ZJ-ES* gene. For example, there may be no significant difference in the expression of the genes that are in the upstream of early senescence regulatory pathway, while the genes in downstream may differentially express due to a series of cascade amplification effects of regulation. In summary, the present localization region is too wide to help us identify the target gene *ZJ-ES*. To narrow the mapping interval, we have constructed a larger mapping population to further map and identify the target gene *ZJ-ES*, and these works will be reported in the future. However, the gene mapping result here shows that 75 predicted genes are different from any reported and cloned rice early senescence genes (Supplementary Table 2), which suggests that *ZJ-ES* is a novel gene regulating early senescence in rice. So *zj-es* may have a new regulation mechanism for early senescence, and, therefore, we performed a whole transcriptome analysis to reveal this new mechanism.

### Autophagy Was Involved in Programmed Cell Death and Early Senescence of *zj-es*

Autophagy is a regulated circulatory metabolic process that is activated to a higher level in response to stress. During this process, the phagosome expands to form the autophagosome with a double-membrane structure, which then binds to the vesicle and transports the cytoplasmic contents or organelles to the vacuole, where they are decomposed and redistributed to the corresponding process (Thompson and Vierstra, 2005; Kwon et al., 2010; van Doorn and Papini, 2013). The role of autophagy in the plant senescent cell is unclear, but extensive degradation of cell components might occur through this mechanism. A lot of autophagy-related genes were identified in our study, such as genes encoding ATG and Rab proteins (Supplementary Figure 9B and Supplementary Table 17). Compared with ZJ22, *OsATG4* (Os04g0682000) and *OsATG8a/b/c/d* (Os07g0512200, Os04g0624000, Os08g0191600, Os11g0100100) were significantly upregulated during *zj-es* senescence, which is consistent with the previous results that *ATG4* and *ATG8* family members were involved in the autophagy process (Pei et al., 2014). Arabidopsis mutants with *atg7* and *atg9* genes knocked out showed an earlier senescence phenotype (Shin et al., 2014), *OsATG7* (Os01g0614900) mutant showed short height, low tiller, and male sterility, and *OsATG7-1* showed a low heading rate and early visible plant senescence (Kurusu et al., 2014; Wada et al., 2015), which was consistent with the characteristics of *zj-es* (Figure 1). In addition, *ATG13* (Os02g0644500, Os11g0162000) is a key factor in autophagy transport and proper senescence (Xie and Klionsky, 2007; Suttangkakul et al., 2011). We found that mi4993-z-mediated ceRNAs upregulated the expression of *OsATG18b* (Os02g0791800) (Figure 8). Previous studies have proved that *AtATG18* is involved in the formation of autophagosomes in Arabidopsis, and the expression of *AtATG18a* is upregulated in the aging state, showing sensitivity to premature senescence (Xiong et al., 2005). Our results also showed that miR11418-z-mediated ceRNAs upregulated the expression of *ATG1* (Os03g0268200) (Figure 8), which was supported by a previous report that *atg1a-atg1b-atg1c-*triple-knockout mutant showed the characteristics of premature plant senescence (Qi et al., 2020). Besides, some *Rab* genes were found to be involved in the occurrence of *zj-es* early senescence. For instance, in the phagosome pathway, *OsRab* family genes were upregulated in the *zj-es* mutant (Supplementary Figure 9B and Supplementary Table 17), while researchers have reported that the expression levels of *OsRab5c* (*Os*03g0666500) and *OsRab7B3* (Os05g0516600) in senescent leaves are higher than those in newborn leaves, and overexpression of *OsRab7B3* can enhance plant senescence in rice (Paranjpe et al., 1990). In the ceRNA regulatory network diagram, we found the mi900-x-mediated upregulation of *RAB7A* (Os01g0227300) (Figure 8). Previous studies have shown that *RAB7* plays an important part in the autophagy pathway and is closely related to the senescence process (Pitakrattananukool et al., 2012), which provides evidence to support our result. In addition, other autophagy-related genes might be also involved in *zj-es* early senescence, such as *OsPLS1* (Os06g0662000), *PI3K* (Os05g0180600), and *SEC61A* (Os08g0254500) (Supplementary Table 17). miR11418-z-mediated ceRNAs upregulated the expression of *SEC61A* in *zj-es* (Figure 8), and SEC61A protein was detected to be located in rice chloroplasts (Figure 10B; Liu et al., 2008), which suggested that *SEC61A* might be involved in the degradation of chloroplasts and the ultimate chlorotic senescence of *zj-es* (Figures 1J–L).

During endocytosis, cargoes were first isolated in a two-membrane structure, and then fused with the endosomes/vacuoles to form autophagosomes, and, finally, decomposable in vacuoles (Li and Vierstra, 2012; Liu and Bassham, 2012). In this study, several genes that regulate vesicle docking and fusion events during endocytosis were significantly upregulated in *zj-es*, including *VPS*, *IST*, *WASH*, and other genes (Supplementary Figure 9C and Supplementary Table 17). Additionally, we found that, in the ceRNAs network diagram, *IST1* (Os04g0398800), *VPS32* (Os09g0267600), and *WASH* (Os06g0136700) were upregulated by miR4993-z, miR5084-x, and miR11418-z-mediated ceRNA, respectively (Figure 8). Overexpression of *Ist1* is necessary for early endosomes to mature into multivesicular endosomes *via* downstream transport routes, which plays an important role in the endocytic pathway (Dimaano et al., 2008; Zhang et al., 2014). As a polymeric protein complex that regulates the dynamics of the endosome tubules, the WASH (Wiskott-Aldrich syndrome homolog) complex acts by binding to the VPS35 protein, targeting it to the endosomes and regulating the activity of membrane mobilization to the endosome-cell surface (Harbour et al., 2012). On the other hand, *Rab* genes also play an important role in endocytic and vesicle transport pathways (Supplementary Figure 9C and Supplementary Table 17). Firstly, the *Rab5* subclass is located in the early endosomal compartment and is involved in the regulation of early endosomal transport by the plasma membrane. For example, *OsRab5a* (Os12g0631100) is involved in the regulation of early endosomal fusion (Gonzalez and Scheller, 1999; Stenmark, 2009). Furthermore, the transition between early-late endosomes could be mediated by Rab transformation, and the upregulated proteins encoded by *Rab5c* (Os03g0666500), *OsRab7* (Os05g0516600), and *OsRab8b* (Os07g0239400) are the main regulatory factors of the endocytic pathway (Chen et al., 2014; El-Esawi and Alayafi, 2019). Additionally, autophagosomes were largely observed in the mesophyll cells of *zj-es* from TEM images (Figure 11). This result was consistent with the expression analysis of autophagy-related genes mentioned above (Supplementary Figure 9 and Supplementary Table 17). Taken together, our results here suggested that autophagy was involved in the early senescence of *zj-es*.

Furthermore, many researchers have proved that autophagy is widely implicated in the regulation of various plant PCD systems (Hayward et al., 2009; Wertman et al., 2012; Li et al., 2016). For example, they found that part of the tapetum retained in *OsATG7-1* mutant was degraded by PCD during late pollen maturation, and they also claimed that tapetal PCD and pollen development required successful Rab7-mediated vacuolar transport in Arabidopsis (Wada et al., 2015; Cui et al., 2017). In addition, endocytosis-related autophagy has been considered to be able to widely induce plant PCD (Faheina-Martins et al., 2012; Shi et al., 2017; Gao et al., 2018). In our results, *zj-es* showed obvious apoptotic plaques in leaves (Figure 1), TB staining, and determination of MDA content of *zj-es* both proved the production of PCD (Figure 12), indicating that various autophagosomes in *zj-es* might trigger early senescence through PCD. Therefore, based on the results here and previous reports, we suggested that autophagy induced the early senescence of *zj-es* by promoting PCD.

### Reactive Oxygen Species Triggered Autophagy and Early Senescence of *zj-es*

Peroxisomes are highly metabolized and self-replicating organelles, which could be acted as β-oxidation by-products of fatty acids to produce ROS (Wang et al., 2016). The significant enrichment of DEGs in the pathway of peroxisome was detected in our study (Figure 7), and a large number of genes whose expressions were increased in the early senescence leaves of *zj-es* are related to the production of ROS (Supplementary Table 17). For example, *OsACX3* (Os06g0354500) and *ACSL* (Os05g0317200, Os01g0681200, Os11g0147000, Os01g0655800) are involved in promoting the β-oxidation of fatty acids with the generation of ROS. *GLO1* (Os03g0786100), *GLO4* (Os07g0152900), *GLO5* (Os07g0616500), and *OsGLO3* (Os04g0623500) also could facilitate the accumulation of H_2_O_2_. *SOD1* (Os03g0351500, Os07g0665200, Os08g0561700) increases the activity of the SOD enzyme that leads to the production of H_2_O_2_. Genes associated with aging, *OsCATA* (Os02g0115700) and *OsCATB* (Os06g0727200), have also been proved to play an important role in the process of H_2_O_2_ metabolism (Lin et al., 2012). As for ceRNA analysis, we found that *SODA* (Os05g0323900) and *ACOX1/3* (Os11g0605500) were upregulated by the miR11418-z-mediated ceRNA network of *zj-es* (Figure 8). Previous studies have shown that the expression of *SODA* is closely related to the change of the ROS level in senescent rice, and the upregulation of *ACOX1/3* is also accompanied by the production of ROS (Wang et al., 2016; You et al., 2020). Moreover, the determination of both the ROS level and the activities of ROS-related enzymes showed that ROS were largely produced and accumulated in the early senescent leaves of *zj-es* (Figure 13), which supported the results of whole-transcriptome analysis.

Various studies have revealed that ROS can induce autophagy and its downstream early senescence in plants. Different researchers have proved that H_2_O_2_ acts as a stimulant to initiate autophagy in *Arabidopsis* (Cuervo, 2004; Xiong et al., 2007a,b). In addition, the accumulation of ROS could activate the intracellular signaling mechanism of endocytosis, and cladesin-mediated endocytosis is involved in regulating the process of yellowing and early senescence of rice mutants at the tillering stage (Chen et al., 2006; Young et al., 2013) that was consistent with the phenotype of *zj-es* leaves at the tillering stage (Figures 1C,D). Research has also suggested that neural cell apoptosis and autophagy can be induced by promoting ROS generation (You et al., 2020). Taken together, ROS might act as a stimulant to trigger the autophagy and early senescence of *zj-es*.

### Molecular Metabolism in Autophagy Was Involved in Early Senescence of *zj-es*

Results of the present study showed that seven metabolic pathways of molecular substance were associated with the early senescence of *zj-es*, and these pathways included glycerolipid metabolism, tyrosine metabolism, fatty acid degradation, glycerophospholipid metabolism, arginine and proline metabolism, phenylalanine metabolism, and ubiquitin-mediated proteolysis (Figure 7B). A large number of studies have revealed that molecular metabolism, especially protein degradation, plays a critical role in the process of plant senescence (Bhalerao et al., 2003; Woo et al., 2016). For example, ubiquitin-mediated protein degradation has been confirmed to be widely involved in plant senescence. Cao et al. (2008) reported that the transcript levels of *UBC1/2*, one of the ubiquitin-conjugating enzyme (E2) genes, increased significantly in leaves and flowers at senescence. Components of the ubiquitin-proteasome complex, including DET1, CUL3, DDB1/2, etc., were also found to act as pivotal factors in the ubiquitin-regulated plant senescence program (Bernhardt et al., 2006; Lyzenga and Stone, 2012; Irigoyen et al., 2014). Moreover, research by Zang et al. (2016) illustrated that ABA biosynthesis was promoted in *OsDET1*-overexpressing transgenic plants with leaf senescence. Our analysis showed that the ubiquitin-mediated proteolysis pathway had a significantly higher enrichment of differential genes, including ubiquitin-conjugating enzyme E2 genes in the senescent leaves of *zj-es* (Supplementary Figure 9D and Supplementary Table 17). Meanwhile, we observed that miR3951-x and miR1510-y-mediated ceRNAs upregulated the expression of *OsDET1* (Os01g0104600) (Figure 8), which means that the corresponding lncRNAs and miRNAs play a regulatory role in early senescence. Thus, we believed that molecular metabolism, especially ubiquitin-mediated protein degradation, might be involved in the early senescence of *zj-es*.

Molecular metabolism, especially protein degradation, is one of the most important events in plant autophagy. Metabonomics suggested that autophagy mutants were involved in the N remobilization, which was associated with arginine metabolism during plant senescence (Masclaux-Daubresse et al., 2014). Furthermore, tapetal autophagy in rice may be involved in the degradation of lipid bodies and the regulation of lipid metabolism during pollen development, and this process is also closely related to peroxisome turnover (Kurusu et al., 2014). Eukaryotic cells use autophagy and the ubiquitin proteasome system (UPS) as their major protein degradation pathways. In this system, damaged or superfluous proteins are ubiquitinated and degraded in the proteasome. Given the evidence that plant ATG1/13 kinase involved in a later step of autophagosome formation undergoes rapid autophagy-dependent degradation in the vacuole during starvation (Suttangkakul et al., 2011; Li et al., 2014), on this basis, researchers also found that the *Arabidopsis thaliana* tumor necrosis factor receptor-associated factor (TRAF) family proteins TRAF1a and TRAF1b help regulate autophagy *via* ubiquitin-mediated autophagic proteolysis, and they are also involved in the ubiquitination degradation of autophagic protein 6 (ATG6) (Qi et al., 2017). Based on the results in this study and analysis above, we then suggested that molecular metabolism in the process of autophagy, especially ubiquitin-mediated protein degradation, was involved in the early senescence of *zj-es*.

### A Possible Model for Triggering Early Senescence of *zj-es*

**FIGURE 14:**
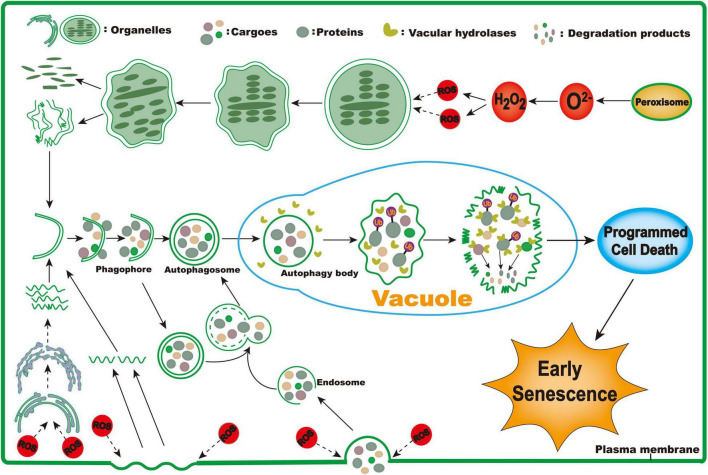
The proposed working model for triggering early senescence in *zj-es*.

Based on the above results, we propose a putative working model for triggering the early senescence of *zj-es* rice (Figure 14). In this model, ROS are largely produced through different redox enzymes in peroxisomes, and these ROS act as a stimulus factor to induce the decomposition of membrane systems, such as chloroplast, endoplasmic reticulum, and plasma membrane. These decomposed membrane structures are used to form phagophores that further expand to form autophagosomes with a double-membrane structure, which encapsulate the substances that need to be metabolized, such as proteins, fatty acids, and lipids. In addition, ROS induce plasma membrane invagination to form endosomes, which take extracellular cargoes into cells. Endosomes fuse with autophagosomes in cells to form new ones. Then, different types of autophagosomes enter the vacuole where the contents in autophagosomes are completely metabolized. For example, the engulfed proteins are eventually degraded under the mediation of ubiquitins. Ultimately, the metabolism and degradation of phagocytic substances lead to cell death and its coupled early senescence of *zj-es*.

## Materials and Methods

### Plant Materials and Growth Conditions

Japonica rice variety ZJ22 was mutagenized by EMS to obtain a mutant library, and an early senescence mutant *zj-es* was isolated from it. All materials were planted in the experimental base of Zhejiang Academy of Agricultural Sciences, Hangzhou, China, from 2019 to 2021. Single plant cultivation was adopted, and the management was the same as field production. The growth status and differences between *zj-es* and wild-type ZJ22 were observed throughout the seedling stage, the tillering stage, and the adult plant stage. Randomly select 10 strains each of the mutant *zj-es* and ZJ22 with consistent growth to investigate agronomic traits, such as plant height, 1,000-grain weight, and grain number per panicle. Take the average value for statistical analysis of the data. The first three leaves were collected from mutant *zj-es* and ZJ22 for whole-transcriptome analysis and designated them as *zj-es*a, *zj-es*b, *zj-es*c (flag leaf, second leaf, third leaf of the mutant), and ZJ22a, ZJ22b, ZJ22c (flag leaf, second leaf, and third leaf of ZJ22), respectively. Each sample contained three biological replicates and leaves from three-leaf positions of three separate plants.

### Histochemical Analysis and Transmission Electron Microscopy

The commercial kits for assaying the contents of GSH, hydrogen peroxide, and MDA, as well as enzyme activities for CAT, SOD, and POD, were all purchased from the Biological Engineering Institute of Nanjing Jiancheng™. Determination experiments were conducted at the six-leaf stage. All determination protocols referred to the manufacturer’s instructions.

Leaves of *zj-es* with apparent necrotic spots and WT in the same growth stage were harvested and stained. Cell death was detected using Trypan blue staining as previously described (Yin et al., 2000). The accumulation of H_2_O_2_ was visually detected using DAB, following the method previously reported (Wang et al., 2013b), after irradiating at 37°C for 10 h; the leaves were then boiled with 90% ethanol to decolorize until the chlorophyll disappeared. After washing with 90% ethanol for 2 h, frozen sections were taken and observed under an optical microscope.

Transmission electron microscope (TEM) observation was performed as described previously (Wang et al., 2013a). Leaves were cut into a target size (1 mm × 3 mm), and 2.5% glutaraldehyde fixation was immersed in a.1-M PBS buffer for fixation. The samples were fixed in OsO_4_, dehydrated in ethanol, and then the samples were put into the embedding agent to infiltrate. The sections (∼70 nm) were cut with diamonds on the ultramicro section (Leica EM UC6). The ultrathin sections were taken on 3-mm copper meshes, stained with uranyl acetate and lead citrate, and, finally, examined with an electron microscope (Hitachi H-7650).

### Mapping of the Gene Locus for the Rice Mutant

Under field conditions, two F2 populations were obtained by self-pollinating the F1 cross between the mutant rice *zj-es* and the wild type rice ZJ22 and, also, the cross of *zj-es* with an indica rice variety, 9311. Genetic analysis was performed on 116 individual plants of *zj-es* × ZJ22 from the F_2_ population and 97 individual plants of *zj-es* × 9311 from the F_2_ population. F2 plants from the *zj-es* × 9311 crosses were used for DNA marker and phenotype segregation analysis to map the gene locus. Total genomic DNA was extracted by the CTAB method. SSR^4^ and InDel (Shen et al., 2004) markers were selected (Supplementary Table 18), and the BSA method was used for gene linkage analysis and gene mapping.

### Libraries Construction, Sequencing, and Screening of lncRNA

Total RNA was extracted with a TRIzol reagent (Invitrogen, CA, United States). The total RNA that passed the quality inspection was used to construct the sequencing libraries. The whole-transcriptome libraries were constructed and sequenced by Gene *Denovo* Biotechnology Co. After total RNA was extracted from the sample, rRNAs were removed to maximize the retention of all coding RNAs and ncRNAs using an rRNA Removal Kit (Epicentre, Madison, WI, United States). After removing ribosomal RNAs, obtained RNAs were randomly interrupted into short fragments under a high temperature, and reverse-transcribed into second-strand cDNAs, which were synthesized with DNA polymerase I, RNase H, dNTP (dUTP instead of dTTP), and a buffer. There were three biological replicates per group and a total of 18 libraries (*zj-es*a, *zj-es*b, *zj-es*c, ZJ22a, ZJ22b, ZJ22c). The digested products were size selected by agarose gel electrophoresis, PCR amplified, and sequenced using an Illumina HiSeqTM4000 (Gene *Denovo* Biotechnology Co.). Raw reads were handled by removing adapter reads and low-quality labels, and all subsequent analyses were performed using clean reads. The small RNA sequencing libraries used Agilent 2100 and qPCR for quality control and computer sequencing.

#### Identification of Differentially Expressed lncRNA and mRNA

The known mRNAs were identified based on rice genome sequence annotation. The assembled transcriptome data were further screened to identify lncRNAs following four steps: (i) the Stringtie (Pertea et al., 2015, 2016) program to predict novel transcripts; (ii) transcripts shorter than 200 bp were first removed; (iii) the protein-coding potential of the new transcripts was removed. For each transcription region, an FPKM value was calculated to quantify its expression abundance and variations, using the StringTie software. DESeq2 (Love et al., 2014) was used to perform RNAs and lncRNAs differential expression analysis between different groups or samples. The genes/transcripts with the parameter of false discovery rate (FDR) below 0.05 and absolute fold change ≥ 2 were considered differentially expressed genes/transcripts.

### Libraries Construction, Sequencing, and Screening of miRNA

A trizol reagent kit (Invitrogen, Carlsbad, CA, United States) was used to extract total RNA. PAGE (polyacrylamide gel electrophoresis) was applied to enrich the RNA molecules of the size range of 18–30 nt and then adding 3′ adapters to enrich 36–44 nt RNAs. The ligation products achieved reverse transcription through PCR amplification, and then PCR products with a size of 140–160 bp were enriched to construct a cDNA library. Sequencing was performed by Gene *Denovo* Biotechnology Co. (Guangzhou, China) using Illumina HiSeq Xten.

To obtain the small RNA clean tags sequence, the small RNA data obtained by preliminary filtering need to be further filtered: (1) removing the low-quality reads (Q-value ≤ 20); (2) removing containing adapters and shorter than 18 nt reads; (3) Removing reads without 3′adapters and containing 5′adapters; (4) removing reads containing ployA in a small RNA fragment. All of the clean data were aligned with small RNAs in the GenBank (Sayers et al., 2021) and Rfam (Kalvari et al., 2018) databases to identify and remove rRNA, scRNA, snoRNA, snRNA, and tRNA, using bowtie (1.1.2) software to identify known miRNAs by aligning clean data with the miRbase database and using MIREAP_v0.2 software to predict novel miRNAs.

#### Identification of Novel miRNA

According to their genome positions and hairpin structures predicted by software mirdeep2, the novel miRNA candidates were identified. The default parameters of software mirdeep2 were as follows: -c: the input file is FASTA format, -h: parse to FASTA format, -i: convert RNA to the DNA alphabet (to map against genome), -j: remove all entries that have a sequence that contains letters other than a, c, g, t, u, n, A, C, G, T, U, or N. -k <seq>: clip 3′ adapter sequence, -l <int>: discard reads shorter than <int>nts, -m: collapse reads, -p <genome>: map to genome (must be indexed by a bowtie build). The genome string must be the prefix of the bowtie index. For instance, if the first indexed file is called h_sapiens_37_asm.1.ebwt, then the prefix is h_sapiens_37_asm. -q: map with one mismatch in the seed (mapping takes longer), -s file: print processed reads to this file, -t file: print read mappings to this file.

#### miRNA Expression Profiles

Total miRNA consists of existing miRNA, known miRNA, and novel miRNA based on their expression in each sample; the miRNA expression level was calculated and normalized to TPM.

#### DEmiRNA Analysis

miRNAs differential expression analysis was performed by the edgeR software between two different groups or samples. We identified miRNAs with a fold change ≥ 2 and *P*-value < 0.05 in a comparison as significant DE miRNAs.

#### Target Gene Prediction of miRNA

The software patmatch (version 1.2) was used to predict target genes. The default parameters were as follows: (1) No more than four mismatches between sRNA/target (G-U bases count as 0.5 mismatches). (2) No more than two adjacent mismatches in the miRNA/target duplex. (3) No adjacent mismatches in positions 2/-12 of the miRNA/target duplex (5\′ of miRNA). (4) No mismatches in positions 10–11 of the miRNA/target duplex. (5) No more than 2.5 mismatches in positions 1/-12 of the miRNA/target duplex (5\′ of miRNA). (6) Minimum free energy (MFE) of the miRNA/target duplex should be \>\ = 74% of the MFE of the miRNA bound to its perfect complement.

### Weighted Gene Co-expression Network Analysis

Weighted gene co-expression network analysis (WGCNA) gene co-expression networks were constructed using the WGCNA package in the R software. After filtering approximately 45% of the genes, gene expression values were imported into WGCNA to construct co-expression modules with default settings except that the power was 8, TOM Type was unsigned, merged cut height was 0.85, and minimum module size was 50. The core DEGs were further divided into eight modules using WGCNA. Module-trait associations were estimated using the correlation between module and specific leaf position expression in the wild type and the mutant. Network visualization for each module was performed using Cytoscape (v3.7.0) (Shannon et al., 2003). The gene co-expression network is a scale-free weighted gene network with multiple nodes connected to different nodes *via* edges. Each node represents a gene, which is connected to a different number of genes. The gene, which is connected to a greater number of genes, is denoted with a bigger size and is more important for its interaction with a large number of genes.

### Target Genes Prediction and Functional Enrichment Analysis

The cist-acting lncRNAs targeted neighboring genes. We searched for 10 kb of coding genes upstream and downstream of all identified lncRNAs and predicted their function. The miRNA-mRNA, mRNA-lncRNA target genes were predicted by patmatch_v1.2 software using small RNA sequencing and RNA-seq data. The filtering conditions (software parameter settings) are as follows: (1) in the targeted complementary relationship between the whole small RNA and the target gene, a maximum of 4 mismatches are allowed (0.5 mismatches for G-U pairing). (2) Continuous mismatch is not allowed. (3) Mismatch is not allowed at the 5′end (2–12 position) of small RNA. (4) The position of 10-11 of small RNA/target is not allowed to mismatch. (5) The position 1–12 of the 5′end of miRNA of small RNA/target allows 2.5 mismatches at most. (6) The MFE (minimum free energy) of small RNA/target must be greater than or equal to 60% of the MFE of perfect combination of miRNA and the target gene.

All DEGs were mapped to GO terms in the GO database,^5^ gene numbers were calculated for every term, and significantly enriched GO terms in DEGs comparing to the genome background were defined by the hypergeometric test. The calculated p-value was gone through FDR Correction, taking FDR ≤ 0.05 as a threshold. GO terms meeting this condition were defined as significantly enriched GO terms in DEGs. KEGG is the major public pathway-related database, and its analysis methods are the same as that in GO analysis.

### Construction of ceRNA Network

According to the ceRNA theory, the method of constructing ceRNA network is as follows: (1) Spearman rank correlation coefficient (SCC) was used to evaluate the expression correlation between mRNA-miRNA or mRNA-lncRNA. Selecting SCC < −0.7 pairs as co-expressed lncRNA-mRNA pairs or mRNA-miRNA pairs, where mRNAs were target genes of miRNA and lncRNA and all RNAs were differentially expressed. (2) Pearson correlation coefficient (PCC) was used to evaluate the correlation between the expressions of lncRNA-mRNA. The pairs with PCC > 0.9 were selected as the co-expressed lncRNA-mRNA pairs, in which both the mRNA and lncRNA in each pair were targeted and co-expressed with common miRNAs. The networks were visualized using Cytoscape_3.3.0 software.

### Quantitative Real-Time PCR

To ensure the reliability of the RNA-Seq results, a selected subset of differently expressed genes was chosen for validation using qRT-PCR in a LightCycler 480 System (Roche, Basel, Switzerland). The reaction mix is consisted of the following: 10-μl final volumes containing 0.2 μl of cDNA, 0.2 μl of each Primer Premier, 5 μl of 2 × U1tra SYBR Mixture, and 4.4-μl RNase-free water. The PCR procedure was as follows: 94°C for 5 min, followed by cycling for 30 rounds at 94°C for 10 s, 60°C for 10 s, and 72°C for 20 s. The experiment was repeated three times using three biological replicates. The relative expression level (fold change) was expressed as 2^–Δ^ ^Δ^ *^Ct^*. The significance level was set to a *P*-value < 0.05. All qRT-PCRs were performed in triplicate.

## Conclusion

Our results show that ROS-triggered autophagy induces the PCD and its coupled early senescence in *zj-es* mutant rice. The *ZJ-ES* gene was mapped in 458 kb-interval between the molecular markers RM15882 and RM293 on Chromosome3. We indicate that the *ZJ-ES* gene is a novel gene for rice early senescence. Meanwhile, we identified 10,085 mRNAs, 1,253 lncRNAs, and 614 miRNAs by using the whole-transcriptome RNA-seq to reveal the different responses of rice to early senescence at different leaf positions. Our results suggested that specific mRNAs and lncRNAs play a role as ceRNAs in response to the onset of early senescence. GO terms and KEGG analysis showed that the mRNA-miRNA-lncRNA competitive regulation network involved peroxisome, autophagy, phagosome, endocytosis, and ubiquitin-mediated proteasome degradation. In general, ROS is the main stimulus signal in the process of early senescence of *zj-es*, which leads to autophagy-mediated PCD and its coupled early senescence. At the same time, metabolism and degradation pathways, such as lipid metabolism and fatty acid degradation, are also involved in early senescence. In conclusion, these results lay a foundation for the study of the response of mRNAs and ncRNAs in rice to early senescence.

## Data Availability Statement

The datasets presented in this study can be found in online repositories. The names of the repository/repositories and accession number(s) can be found below: National Center for Biotechnology Information (NCBI) BioProject database under accession number PRJNA793760. The main scripts of the data analysis can be found below: https://github.com/jiasun8690/Whole-transcriptome_RNA.

## Author Contributions

YY and JC contributed to conceptualization, validation, and project administration. JS and YY contributed to formal analysis and methodology, data curation, and writing the original draft. JS contributed to software. JS, YY, WL, XC, YZ, JL, YS, XW, JZ, CYu, CYa, and BZ investigated the study. YY, SY, and JS contributed to resources. JS, YY, and JC contributed to writing, reviewing, and editing the manuscript. All authors have read the final manuscript and approved the submission.

## Conflict of Interest

The authors declare that the research was conducted in the absence of any commercial or financial relationships that could be construed as a potential conflict of interest.

## Publisher’s Note

All claims expressed in this article are solely those of the authors and do not necessarily represent those of their affiliated organizations, or those of the publisher, the editors and the reviewers. Any product that may be evaluated in this article, or claim that may be made by its manufacturer, is not guaranteed or endorsed by the publisher.

## Footnotes

1
https://rapdb.dna.affrc.go.jp/

2
http://www.rproject.org/

3
http://cpc.cbi.pku.edu.cn/

4
http://www.gramene.org/

5
http://www.geneontology.org/

## References

[B1] Alonso J. M. Hirayama T. Roman G. Nourizadeh S. Ecker J. R. (1999). EIN2, a bifunctional transducer of ethylene and stress responses in *Arabidopsis*. *Science* 284 2148–2152. 10.1126/science.284.5423.2148 10381874

[B2] Balazadeh S. Jaspert N. Arif M. Mueller-Roeber B. Maurino V. G. (2012). Expression of ROS-responsive genes and transcription factors after metabolic formation of H2O2 in chloroplasts. *Front. Plant Sci.* 3:234. 10.3389/fpls.2012.00234 23125844 PMC3485569

[B3] Bartel D. P. (2004). MicroRNAs: genomics, biogenesis, mechanism, and function. *Cell* 116 281–297. 10.1016/s0092-8674(04)00045-5 14744438

[B4] Ben Amor B. Wirth S. Merchan F. Laporte P. d’Aubenton-Carafa Y. Hirsch J. (2009). Novel long non-protein coding RNAs involved in *Arabidopsis* differentiation and stress responses. *Genome Res.* 19 57–69. 10.1101/gr.080275.108 18997003 PMC2612962

[B5] Bernhardt A. Lechner E. Hano P. Schade V. Dieterle M. Anders M. (2006). CUL4 associates with DDB1 and DET1 and its downregulation affects diverse aspects of development in *Arabidopsis* thaliana. *Plant J.* 47 591–603. 10.1111/j.1365-313X.2006.02810.x 16792691

[B6] Bhalerao R. Keskitalo J. Sterky F. Erlandsson R. Bjorkbacka H. Birve S. J. (2003). Gene expression in autumn leaves. *Plant Physiol.* 131 430–442. 10.1104/pp.012732 12586868 PMC166820

[B7] Bieker S. Riester L. Stahl M. Franzaring J. Zentgraf U. (2012). Senescence-specific alteration of hydrogen peroxide levels in *Arabidopsis* thaliana and oilseed rape spring variety *Brassica napus* L. cv. *Mozart. J. Integr. Plant Biol.* 54 540–554. 10.1111/j.1744-7909.2012.01147.x 22805117

[B8] Buono R. A. Hudecek R. Nowack M. K. (2019). Plant proteases during developmental programmed cell death. *J. Exp. Bot.* 70 2097–2112. 10.1093/jxb/erz072 30793182 PMC7612330

[B9] Cao Y. Dai Y. Cui S. Ma L. (2008). Histone H2B monoubiquitination in the chromatin of FLOWERING LOCUS C regulates flowering time in Arabidopsis. *Plant Cell* 20, 2586–2602. 10.1105/tpc.108.062760 18849490 PMC2590739

[B10] Chekanova J. A. (2015). Long non-coding RNAs and their functions in plants. *Curr. Opin. Plant Biol.* 27 207–216. 10.1016/j.pbi.2015.08.003 26342908

[B11] Chen L. L. (2016). Linking long noncoding RNA localization and function. *Trends Biochem. Sci.* 41 761–772. 10.1016/j.tibs.2016.07.003 27499234

[B12] Chen P. I. Schauer K. Kong C. Harding A. R. Goud B. Stahl P. D. (2014). Rab5 isoforms orchestrate a “division of labor” in the endocytic network. Rab5C modulates rac-mediated cell motility. *PLoS One* 9:e90384. 10.1371/journal.pone.0090384 24587345 PMC3938722

[B13] Chen S. Zhou Y. Chen Y. Gu J. (2018). fastp: an ultra-fast all-in-one FASTQ preprocessor. *Bioinformatics* 34 i884–i890. 10.1093/bioinformatics/bty560 30423086 PMC6129281

[B14] Chen X. (2004). A microRNA as a translational repressor of APETALA2 in *Arabidopsis* flower development. *Science* 303 2022–2025. 10.1126/science.1088060 12893888 PMC5127708

[B15] Chen Y. Xu Y. Luo W. Li W. Chen N. Zhang D. (2013). The F-box protein OsFBK12 targets OsSAMS1 for degradation and affects pleiotropic phenotypes, including leaf senescence, in rice. *Plant Physiol.* 163 1673–1685. 10.1104/pp.113.224527 24144792 PMC3850201

[B16] Chen Z. Krmar R. T. Dada L. Efendiev R. Leibiger I. B. Pedemonte C. H. (2006). Phosphorylation of adaptor protein-2 mu2 is essential for Na+,K+-ATPase endocytosis in response to either G protein-coupled receptor or reactive oxygen species. *Am. J. Respir. Cell Mol. Biol.* 35 127–132. 10.1165/rcmb.2006-0044OC 16498080 PMC2658693

[B17] Cuervo A. M. (2004). Autophagy: in sickness and in health. *Trends Cell Biol.* 14 70–77. 10.1016/j.tcb.2003.12.002 15102438

[B18] Cui Y. Zhao Q. Xie H. T. Wong W. S. Wang X. Gao C. (2017). Monensin sensitivity1 (MON1)/calcium caffeine zinc sensitivity1 (CCZ1)-mediated Rab7 activation regulates tapetal Programmed cell death and pollen development. *Plant Physiol.* 173 206–218. 10.1104/pp.16.00988 27799422 PMC5210713

[B19] Dimaano C. Jones C. B. Hanono A. Curtiss M. Babst M. (2008). Ist1 regulates Vps4 localization and assembly. *Mol. Biol. Cell* 19 465–474. 10.1091/mbc.e07-08-0747 18032582 PMC2230601

[B20] Ding J. Lu Q. Ouyang Y. Mao H. Zhang P. Yao J. (2012). A long noncoding RNA regulates photoperiod-sensitive male sterility, an essential component of hybrid rice. *Proc. Natl. Acad. Sci. U. S. A.* 109 2654–2659. 10.1073/pnas.1121374109 22308482 PMC3289353

[B21] Drake R. John I. Farrell A. Cooper W. Schuch W. Grierson D. (1996). Isolation and analysis of cDNAs encoding tomato cysteine proteases expressed during leaf senescence. *Plant Mol. Biol.* 30 755–767. 10.1007/BF00019009 8624407

[B22] El-Esawi M. A. Alayafi A. A. (2019). Overexpression of rice rab7 gene improves drought and heat tolerance and increases grain yield in rice (*Oryza sativa* L.). *Genes* 10:56. 10.3390/genes10010056 30658457 PMC6357162

[B23] Evans I. M. Rus A. M. Belanger E. M. Kimoto M. Brusslan J. A. (2010). Dismantling of *Arabidopsis* thaliana mesophyll cell chloroplasts during natural leaf senescence. *Plant Biol.* 12 1–12. 10.1111/j.1438-8677.2009.00206.x 20653883 PMC4383266

[B24] Faheina-Martins G. V. da Silveira A. L. Cavalcanti B. C. Ramos M. V. Moraes M. O. Pessoa C. (2012). Antiproliferative effects of lectins from *Canavalia ensiformis* and *Canavalia brasiliensis* in human leukemia cell lines. *Toxicol In Vitro* 26 1161–1169. 10.1016/j.tiv.2012.06.017 22776218

[B25] Finkelstein R. R. Rock C. D. (2002). Abscisic acid biosynthesis and response. *Arabidopsis Book* 1:e0058. 10.1199/tab.0058 22303212 PMC3243367

[B26] Franco-Zorrilla J. M. Valli A. Todesco M. Mateos I. Puga M. I. Rubio-Somoza I. (2007). Target mimicry provides a new mechanism for regulation of microRNA activity. *Nat. Genet.* 39 1033–1037. 10.1038/ng2079 17643101

[B27] Gao F. Mei X. Li Y. Guo J. Shen Y. (2021). Update on the roles of polyamines in fleshy fruit ripening, senescence, and quality. *Front. Plant Sci.* 12:610313. 10.3389/fpls.2021.610313 33664757 PMC7922164

[B28] Gao X. Ruan X. Sun Y. Wang X. Feng B. (2018). BAKing up to survive a battle: functional dynamics of BAK1 in plant programmed cell death. *Front. Plant Sci.* 9:1913. 10.3389/fpls.2018.01913 30671069 PMC6331536

[B29] Gonzalez L. Jr. Scheller R. H. (1999). Regulation of membrane trafficking: structural insights from a Rab/effector complex. *Cell* 96 755–758. 10.1016/s0092-8674(00)80585-1 10102263

[B30] Harbour M. E. Breusegem S. Y. Seaman M. N. (2012). Recruitment of the endosomal WASH complex is mediated by the extended ‘tail’ of Fam21 binding to the retromer protein Vps35. *Biochem. J.* 442 209–220. 10.1042/BJ20111761 22070227

[B31] Hayward A. P. Tsao J. Dinesh-Kumar S. P. (2009). Autophagy and plant innate immunity: defense through degradation. *Semin. Cell Dev. Biol.* 20 1041–1047. 10.1016/j.semcdb.2009.04.012 19406248

[B32] Hirayama T. Shinozaki K. (2007). Perception and transduction of abscisic acid signals: keys to the function of the versatile plant hormone ABA. *Trends Plant Sci.* 12 343–351. 10.1016/j.tplants.2007.06.013 17629540

[B33] Hong Y. Zhang Y. Sinumporn S. Yu N. Zhan X. Shen X. (2018). Premature leaf senescence 3, encoding a methyltransferase, is required for melatonin biosynthesis in rice. *Plant J.* 95, 877–891. 10.1111/tpj.13995 29901843

[B34] Hu J. Jin J. Qian Q. Huang K. Ding Y. (2016). Small RNA and degradome profiling reveals miRNA regulation in the seed germination of ancient eudicot *Nelumbo nucifera*. *BMC Genomics* 17:684. 10.1186/s12864-016-3032-4 27565736 PMC5002175

[B35] Hudson D. Guevara D. R. Hand A. J. Xu Z. Hao L. Chen X. (2013). Rice cytokinin GATA transcription factor1 regulates chloroplast development and plant architecture. *Plant Physiol.* 162 132–144. 10.1104/pp.113.217265 23548780 PMC3641198

[B36] Irigoyen M. L. Iniesto E. Rodriguez L. Puga M. I. Yanagawa Y. Pick E. (2014). Targeted degradation of abscisic acid receptors is mediated by the ubiquitin ligase substrate adaptor DDA1 in *Arabidopsis*. *Plant Cell* 26 712–728. 10.1105/tpc.113.122234 24563205 PMC3967035

[B37] Jiang H. Li M. Liang N. Yan H. Wei Y. Xu X. (2007). Molecular cloning and function analysis of the stay green gene in rice. *Plant J.* 52 197–209. 10.1111/j.1365-313X.2007.03221.x 17714430

[B38] Jiao B. B. Wang J. J. Zhu X. D. Zeng L. J. Li Q. He Z. H. (2012). A novel protein RLS1 with NB-ARM domains is involved in chloroplast degradation during leaf senescence in rice. *Mol. Plant* 5 205–217. 10.1093/mp/ssr081 21980143

[B39] Jones D. P. (2006). Redefining oxidative stress. *Antioxid. Redox. Signal* 8 1865–1879. 10.1089/ars.2006.8.1865 16987039

[B40] Kalvari I. Nawrocki E. P. Argasinska J. Quinones-Olvera N. Finn R. D. Bateman A. (2018). Non-Coding RNA analysis using the Rfam database. *Curr. Protoc. Bioinformatics* 62:e51. 10.1002/cpbi.51 29927072 PMC6754622

[B41] Kang K. Kim Y. S. Park S. Back K. (2009). Senescence-induced serotonin biosynthesis and its role in delaying senescence in rice leaves. *Plant Physiol.* 150 1380–1393. 10.1104/pp.109.138552 19439571 PMC2705024

[B42] Kim D. H. Sung S. (2017). Vernalization-triggered intragenic chromatin loop formation by long noncoding RNAs. *Dev. Cell* 40 302–312e304. 10.1016/j.devcel.2016.12.021 28132848 PMC5303624

[B43] Kim J. Woo H. R. Nam H. G. (2016). Toward systems understanding of leaf senescence: an integrated multi-omics perspective on leaf senescence research. *Mol. Plant* 9 813–825. 10.1016/j.molp.2016.04.017 27174403

[B44] Kim J. H. Woo H. R. Kim J. Lim P. O. Lee I. C. Choi S. H. (2009). Trifurcate feed-forward regulation of age-dependent cell death involving miR164 in *Arabidopsis*. *Science* 323 1053–1057. 10.1126/science.1166386 19229035

[B45] Kong L. Zhang Y. Ye Z. Q. Liu X. Q. Zhao S. Q. Wei L. (2007). CPC: assess the protein-coding potential of transcripts using sequence features and support vector machine. *Nucleic Acids Res.* 35 W345–W349. 10.1093/nar/gkm391 17631615 PMC1933232

[B46] Krieger-Liszkay A. Krupinska K. Shimakawa G. (2019). The impact of photosynthesis on initiation of leaf senescence. *Physiol. Plant* 166 148–164. 10.1111/ppl.12921 30629302

[B47] Kurusu T. Koyano T. Hanamata S. Kubo T. Noguchi Y. Yagi C. (2014). OsATG7 is required for autophagy-dependent lipid metabolism in rice postmeiotic anther development. *Autophagy* 10 878–888. 10.4161/auto.28279 24674921 PMC5119067

[B48] Kusaba M. Ito H. Morita R. Iida S. Sato Y. Fujimoto M. (2007). Rice NON-YELLOW COLORING1 is involved in light-harvesting complex II and grana degradation during leaf senescence. *Plant Cell* 19 1362–1375. 10.1105/tpc.106.042911 17416733 PMC1913755

[B49] Kusumi K. Sakata C. Nakamura T. Kawasaki S. Yoshimura A. Iba K. (2011). A plastid protein NUS1 is essential for build-up of the genetic system for early chloroplast development under cold stress conditions. *Plant J.* 68 1039–1050. 10.1111/j.1365-313X.2011.04755.x 21981410

[B50] Kwon S. I. Cho H. J. Jung J. H. Yoshimoto K. Shirasu K. Park O. K. (2010). The Rab GTPase RabG3b functions in autophagy and contributes to tracheary element differentiation in *Arabidopsis*. *Plant J.* 64 151–164. 10.1111/j.1365-313X.2010.04315.x 20659276

[B51] Lee R. H. Lin M. C. Chen S. C. (2004). A novel alkaline alpha-galactosidase gene is involved in rice leaf senescence. *Plant Mol Biol* 55 281–295. 10.1007/s11103-004-0641-0 15604681

[B52] Li F. Chung T. Vierstra R. D. (2014). AUTOPHAGY-RELATED11 plays a critical role in general autophagy- and senescence-induced mitophagy in *Arabidopsis*. *Plant Cell* 26 788–807. 10.1105/tpc.113.120014 24563201 PMC3967041

[B53] Li F. Vierstra R. D. (2012). Autophagy: a multifaceted intracellular system for bulk and selective recycling. *Trends Plant Sci.* 17 526–537. 10.1016/j.tplants.2012.05.006 22694835

[B54] Li Y. Kabbage M. Liu W. Dickman M. B. (2016). Aspartyl protease-mediated cleavage of BAG6 is necessary for autophagy and fungal resistance in plants. *Plant Cell* 28 233–247. 10.1105/tpc.15.00626 26739014 PMC4746679

[B55] Li Z. An X. Zhu T. Yan T. Wu S. Tian Y. (2019). Discovering and constructing ceRNA-miRNA-Target gene regulatory networks during anther development in maize. *Int. J. Mol. Sci.* 20:3480. 10.3390/ijms20143480 31311189 PMC6678786

[B56] Li Z. Peng J. Wen X. Guo H. (2013). Ethylene-insensitive3 is a senescence-associated gene that accelerates age-dependent leaf senescence by directly repressing miR164 transcription in *Arabidopsis*. *Plant Cell* 25 3311–3328. 10.1105/tpc.113.113340 24064769 PMC3809534

[B57] Liang C. Wang Y. Zhu Y. Tang J. Hu B. Liu L. (2014). OsNAP connects abscisic acid and leaf senescence by fine-tuning abscisic acid biosynthesis and directly targeting senescence-associated genes in rice. *Proc. Natl. Acad. Sci. U. S. A.* 111 10013–10018. 10.1073/pnas.1321568111 24951508 PMC4103337

[B58] Lim P. O. Kim H. J. Nam H. G. (2007). Leaf senescence. *Annu. Rev. Plant Biol.* 58 115–136. 10.1146/annurev.arplant.57.032905.105316 17177638

[B59] Lin A. Wang Y. Tang J. Xue P. Li C. Liu L. (2012). Nitric oxide and protein S-nitrosylation are integral to hydrogen peroxide-induced leaf cell death in rice. *Plant Physiol.* 158 451–464. 10.1104/pp.111.184531 22106097 PMC3252116

[B60] Liu J. Wang H. Chua N. H. (2015a). Long noncoding RNA transcriptome of plants. *Plant Biotechnol J.* 13 319–328. 10.1111/pbi.12336 25615265

[B61] Liu L. Zhou Y. Zhou G. Ye R. Zhao L. Li X. (2008). Identification of early senescence-associated genes in rice flag leaves. *Plant Mol. Biol.* 67 37–55. 10.1007/s11103-008-9300-1 18330710

[B62] Liu X. Hao L. Li D. Zhu L. Hu S. (2015b). Long non-coding RNAs and their biological roles in plants. *Genomics Proteomics Bioinformatics* 13 137–147. 10.1016/j.gpb.2015.02.003 25936895 PMC4563214

[B63] Liu Y. Bassham D. C. (2012). Autophagy: pathways for self-eating in plant cells. *Annu. Rev. Plant Biol.* 63 215–237. 10.1146/annurev-arplant-042811-105441 22242963

[B64] Love M. I. Huber W. Anders S. (2014). Moderated estimation of fold change and dispersion for RNA-seq data with DESeq2. *Genome. Biol.* 15:550. 10.1186/s13059-014-0550-8 25516281 PMC4302049

[B65] Lyzenga W. J. Stone S. L. (2012). Abiotic stress tolerance mediated by protein ubiquitination. *J. Exp. Bot.* 63 599–616. 10.1093/jxb/err310 22016431

[B66] Masclaux-Daubresse C. Clement G. Anne P. Routaboul J. M. Guiboileau A. Soulay F. (2014). Stitching together the multiple dimensions of autophagy using metabolomics and transcriptomics reveals impacts on metabolism, development, and plant responses to the environment in *Arabidopsis*. *Plant Cell* 26 1857–1877. 10.1105/tpc.114.124677 24808053 PMC4079355

[B67] Meng X. Zhang P. Chen Q. Wang J. Chen M. (2018). Identification and characterization of ncRNA-associated ceRNA networks in *Arabidopsis* leaf development. *BMC Genomics* 19:607. 10.1186/s12864-018-4993-2 30103673 PMC6090674

[B68] Millar A. A. Gubler F. (2005). The *Arabidopsis* GAMYB-like genes, MYB33 and MYB65, are microRNA-regulated genes that redundantly facilitate anther development. *Plant Cell* 17 705–721. 10.1105/tpc.104.027920 15722475 PMC1069693

[B69] Mittler R. Vanderauwera S. Gollery M. Van Breusegem F. (2004). Reactive oxygen gene network of plants. *Trends Plant Sci.* 9 490–498. 10.1016/j.tplants.2004.08.009 15465684

[B70] Mochizuki-Kawai H. Niki T. Shibuya K. Ichimura K. (2015). Programmed cell death progresses differentially in epidermal and mesophyll cells of lily petals. *PLoS One* 10:e0143502. 10.1371/journal.pone.0143502 26605547 PMC4659684

[B71] Paranjpe P. Patki P. Patwardhan B. (1990). Ayurvedic treatment of obesity: a randomised double-blind, placebo-controlled clinical trial. *J. Ethnopharmacol.* 29 1–11. 10.1016/0378-8741(90)90092-8 2278549

[B72] Pei D. Zhang W. Sun H. Wei X. Yue J. Wang H. (2014). Identification of autophagy-related genes ATG4 and ATG8 from wheat (*Triticum aestivum* L.) and profiling of their expression patterns responding to biotic and abiotic stresses. *Plant Cell Rep.* 33 1697–1710. 10.1007/s00299-014-1648-x 24996626

[B73] Pertea M. Kim D. Pertea G. M. Leek J. T. Salzberg S. L. (2016). Transcript-level expression analysis of RNA-seq experiments with HISAT, stringtie and ballgown. *Nat. Protoc.* 11 1650–1667. 10.1038/nprot.2016.095 27560171 PMC5032908

[B74] Pertea M. Pertea G. M. Antonescu C. M. Chang T. C. Mendell J. T. Salzberg S. L. (2015). StringTie enables improved reconstruction of a transcriptome from RNA-seq reads. *Nat. Biotechnol* 33 290–295. 10.1038/nbt.3122 25690850 PMC4643835

[B75] Petrov V. Hille J. Mueller-Roeber B. Gechev T. S. (2015). ROS-mediated abiotic stress-induced programmed cell death in plants. *Front. Plant Sci.* 6:69. 10.3389/fpls.2015.00069 25741354 PMC4332301

[B76] Pitakrattananukool S. Kawakatsu T. Anuntalabhochai S. Takaiwa F. (2012). Overexpression of OsRab7B3, a small GTP-binding protein gene, enhances leaf senescence in transgenic rice. *Biosci. Biotechnol. Biochem.* 76 1296–1302. 10.1271/bbb.120050 22785493

[B77] Qi H. Li J. Xia F. N. Chen J. Y. Lei X. Han M. Q. (2020). *Arabidopsis* SINAT proteins control autophagy by mediating ubiquitylation and degradation of ATG13. *Plant Cell* 32 263–284. 10.1105/tpc.19.00413 31732704 PMC6961628

[B78] Qi H. Xia F. N. Xie L. J. Yu L. J. Chen Q. F. Zhuang X. H. (2017). TRAF family proteins regulate autophagy dynamics by modulating autophagy protein6 stability in *Arabidopsis*. *Plant Cell* 29 890–911. 10.1105/tpc.17.00056 28351989 PMC5435438

[B79] Robert-Seilaniantz A. Grant M. Jones J. D. (2011). Hormone crosstalk in plant disease and defense: more than just jasmonate-salicylate antagonism. *Annu. Rev. Phytopathol.* 49 317–343. 10.1146/annurev-phyto-073009-114447 21663438

[B80] Salmena L. Poliseno L. Tay Y. Kats L. Pandolfi P. P. (2011). A ceRNA hypothesis: the rosetta stone of a hidden RNA language? *Cell* 146 353–358. 10.1016/j.cell.2011.07.014 21802130 PMC3235919

[B81] Sayers E. W. Cavanaugh M. Clark K. Pruitt K. D. Schoch C. L. Sherry S. T. (2021). Genbank. *Nucleic. Acids Res.* 49 D92–D96. 10.1093/nar/gkaa1023 33196830 PMC7778897

[B82] Schommer C. Palatnik J. F. Aggarwal P. Chetelat A. Cubas P. Farmer E. E. (2008). Control of jasmonate biosynthesis and senescence by miR319 targets. *PLoS Biol.* 6:e230. 10.1371/journal.pbio.0060230 18816164 PMC2553836

[B83] Schottler M. A. Thiele W. Belkius K. Bergner S. V. Flugel C. Wittenberg G. (2017). The plastid-encoded PsaI subunit stabilizes photosystem I during leaf senescence in tobacco. *J. Exp. Bot.* 68 1137–1155. 10.1093/jxb/erx009 28180288 PMC5429015

[B84] Seo J. S. Sun H. X. Park B. S. Huang C. H. Yeh S. D. Jung C. (2017). ELF18-INDUCED LONG-NONCODING RNA associates with mediator to enhance expression of innate immune response genes in *Arabidopsis*. *Plant Cell* 29 1024–1038. 10.1105/tpc.16.00886 28400491 PMC5466027

[B85] Shannon P. Markiel A. Ozier O. Baliga N. S. Wang J. T. Ramage D. (2003). Cytoscape: a software environment for integrated models of biomolecular interaction networks. *Genome. Res.* 13 2498–2504. 10.1101/gr.1239303 14597658 PMC403769

[B86] Shen Y. J. Jiang H. Jin J. P. Zhang Z. B. Xi B. He Y. Y. (2004). Development of genome-wide DNA polymorphism database for map-based cloning of rice genes. *Plant Physiol.* 135 1198–1205. 10.1104/pp.103.038463 15266053 PMC519040

[B87] Shi Z. Li W. W. Tang Y. Cheng L. J. (2017). A novel molecular model of plant lectin-induced programmed cell death in cancer. *Biol. Pharm. Bull.* 40 1625–1629. 10.1248/bpb.b17-00363 28768938

[B88] Shin K. D. Lee H. N. Chung T. (2014). A revised assay for monitoring autophagic flux in *Arabidopsis* thaliana reveals involvement of AUTOPHAGY-RELATED9 in autophagy. *Mol. Cells* 37 399–405. 10.14348/molcells.2014.0042 24805779 PMC4044311

[B89] Singh S. Giri M. K. Singh P. K. Siddiqui A. Nandi A. K. (2013). Down-regulation of OsSAG12-1 results in enhanced senescence and pathogen-induced cell death in transgenic rice plants. *J. Biosci.* 38 583–592. 10.1007/s12038-013-9334-7 23938390

[B90] Smykowski A. Zimmermann P. Zentgraf U. (2010). G-Box binding factor1 reduces CATALASE2 expression and regulates the onset of leaf senescence in *Arabidopsis*. *Plant Physiol.* 153 1321–1331. 10.1104/pp.110.157180 20484024 PMC2899923

[B91] Stenmark H. (2009). Rab GTPases as coordinators of vesicle traffic. *Nat. Rev. Mol. Cell Biol.* 10 513–525. 10.1038/nrm2728 19603039

[B92] Sun L. Luo H. Bu D. Zhao G. Yu K. Zhang C. (2013). Utilizing sequence intrinsic composition to classify protein-coding and long non-coding transcripts. *Nucleic. Acids Res.* 41:e166. 10.1093/nar/gkt646 23892401 PMC3783192

[B93] Suttangkakul A. Li F. Chung T. Vierstra R. D. (2011). The ATG1/ATG13 protein kinase complex is both a regulator and a target of autophagic recycling in *Arabidopsis*. *Plant Cell* 23 3761–3779. 10.1105/tpc.111.090993 21984698 PMC3229148

[B94] Thomas H. (2013). Senescence, ageing and death of the whole plant. *New Phytol.* 197 696–711. 10.1111/nph.12047 23176101

[B95] Thompson A. R. Vierstra R. D. (2005). Autophagic recycling: lessons from yeast help define the process in plants. *Curr. Opin. Plant Biol.* 8 165–173. 10.1016/j.pbi.2005.01.013 15752997

[B96] van Doorn W. G. Papini A. (2013). Ultrastructure of autophagy in plant cells: a review. *Autophagy* 9 1922–1936. 10.4161/auto.26275 24145319

[B97] Vanacker H. Sandalio L. Jimenez A. Palma J. M. Corpas F. J. Meseguer V. (2006). Roles for redox regulation in leaf senescence of pea plants grown on different sources of nitrogen nutrition. *J. Exp. Bot.* 57 1735–1745. 10.1093/jxb/erl012 16760420

[B98] Wada S. Hayashida Y. Izumi M. Kurusu T. Hanamata S. Kanno K. (2015). Autophagy supports biomass production and nitrogen use efficiency at the vegetative stage in rice. *Plant Physiol.* 168 60–73. 10.1104/pp.15.00242 25786829 PMC4424030

[B99] Wang F. Liu J. Zhou L. Pan G. Li Z. Zaidi S. H. (2016). Senescence-specific change in ROS scavenging enzyme activities and regulation of various SOD isozymes to ROS levels in psf mutant rice leaves. *Plant Physiol. Biochem.* 109 248–261. 10.1016/j.plaphy.2016.10.005 27756006

[B100] Wang T. Yang B. Guan Q. Chen X. Zhong Z. Huang W. (2019a). Transcriptional regulation of *Lonicera japonica* Thunb. during flower development as revealed by comprehensive analysis of transcription factors. *BMC Plant Biol.* 19:198. 10.1186/s12870-019-1803-1 31088368 PMC6518806

[B101] Wang W. Paschalidis K. Feng J. C. Song J. Liu J. H. (2019b). Polyamine catabolism in plants: a universal process with diverse functions. *Front. Plant Sci.* 10:561. 10.3389/fpls.2019.00561 31134113 PMC6513885

[B102] Wang Y. Fan X. Lin F. He G. Terzaghi W. Zhu D. (2014). *Arabidopsis* noncoding RNA mediates control of photomorphogenesis by red light. *Proc. Natl. Acad. Sci. U. S. A.* 111 10359–10364. 10.1073/pnas.1409457111 24982146 PMC4104870

[B103] Wang Y. Yu B. Zhao J. Guo J. Li Y. Han S. (2013a). Autophagy contributes to leaf starch degradation. *Plant Cell* 25 1383–1399. 10.1105/tpc.112.108993 23564204 PMC3663275

[B104] Wang Y. Zhang Y. Wang Z. Zhang X. Yang S. (2013b). A missense mutation in CHS1, a TIR-NB protein, induces chilling sensitivity in *Arabidopsis*. *Plant J.* 75 553–565. 10.1111/tpj.12232 23651299

[B105] Wertman J. Lord C. E. Dauphinee A. N. Gunawardena A. H. (2012). The pathway of cell dismantling during programmed cell death in lace plant (Aponogeton madagascariensis) leaves. *BMC Plant Biol.* 12:115. 10.1186/1471-2229-12-115 22828052 PMC3466136

[B106] Winterbourn C. C. Metodiewa D. (1994). The reaction of superoxide with reduced glutathione. *Arch. Biochem. Biophys.* 314 284–290. 10.1006/abbi.1994.1444 7979367

[B107] Woo H. R. Kim H. J. Nam H. G. Lim P. O. (2013). Plant leaf senescence and death - regulation by multiple layers of control and implications for aging in general. *J. Cell Sci.* 126(Pt 21), 4823–4833. 10.1242/jcs.109116 24144694

[B108] Woo H. R. Koo H. J. Kim J. Jeong H. Yang J. O. Lee I. H. (2016). Programming of plant leaf senescence with temporal and inter-organellar coordination of transcriptome in *Arabidopsis*. *Plant Physiol.* 171 452–467. 10.1104/pp.15.01929 26966169 PMC4854694

[B109] Xie Z. Klionsky D. J. (2007). Autophagosome formation: core machinery and adaptations. *Nat. Cell Biol.* 9 1102–1109. 10.1038/ncb1007-1102 17909521

[B110] Xiong Y. Contento A. L. Bassham D. C. (2005). AtATG18a is required for the formation of autophagosomes during nutrient stress and senescence in *Arabidopsis* thaliana. *Plant J.* 42 535–546. 10.1111/j.1365-313X.2005.02397.x 15860012

[B111] Xiong Y. Contento A. L. Bassham D. C. (2007a). Disruption of autophagy results in constitutive oxidative stress in *Arabidopsis*. *Autophagy* 3 257–258. 10.4161/auto.3847 17312382

[B112] Xiong Y. Contento A. L. Nguyen P. Q. Bassham D. C. (2007b). Degradation of oxidized proteins by autophagy during oxidative stress in *Arabidopsis*. *Plant Physiol.* 143 291–299. 10.1104/pp.106.092106 17098847 PMC1761971

[B113] Xu X. W. Zhou X. H. Wang R. R. Peng W. L. An Y. Chen L. L. (2016). Functional analysis of long intergenic non-coding RNAs in phosphate-starved rice using competing endogenous RNA network. *Sci. Rep.* 6:20715. 10.1038/srep20715 26860696 PMC4748279

[B114] Yamatani H. Sato Y. Masuda Y. Kato Y. Morita R. Fukunaga K. (2013). NYC4, the rice ortholog of *Arabidopsis* THF1, is involved in the degradation of chlorophyll - protein complexes during leaf senescence. *Plant J.* 74 652–662. 10.1111/tpj.12154 23432654

[B115] Yan J. Chen Q. Cui X. Zhao P. Gao S. Yang B. (2021). Ectopic overexpression of a membrane-tethered transcription factor gene NAC60 from oilseed rape positively modulates programmed cell death and age-triggered leaf senescence. *Plant J.* 105 600–618. 10.1111/tpj.15057 33119146

[B116] Yan J. Zhang H. Zheng Y. Ding Y. (2015). Comparative expression profiling of miRNAs between the cytoplasmic male sterile line MeixiangA and its maintainer line MeixiangB during rice anther development. *Planta* 241 109–123. 10.1007/s00425-014-2167-2 25228384

[B117] Yang Z. Yang C. Wang Z. Yang Z. Chen D. Wu Y. (2019). LncRNA expression profile and ceRNA analysis in tomato during flowering. *PLoS One* 14:e0210650. 10.1371/journal.pone.0210650 30653557 PMC6336255

[B118] Yin L. L. Xue H. W. (2012). The MADS29 transcription factor regulates the degradation of the nucellus and the nucellar projection during rice seed development. *Plant Cell* 24 1049–1065. 10.1105/tpc.111.094854 22408076 PMC3336122

[B119] Yin Z. Chen J. Zeng L. Goh M. Leung H. Khush G. S. (2000). Characterizing rice lesion mimic mutants and identifying a mutant with broad-spectrum resistance to rice blast and bacterial blight. *Mol. Plant Microbe. Interact.* 13 869–876. 10.1094/MPMI.2000.13.8.869 10939258

[B120] You L. Chen J. Liu W. Xiang Q. Luo Z. Wang W. (2020). Enterovirus 71 induces neural cell apoptosis and autophagy through promoting ACOX1 downregulation and ROS generation. *Virulence* 11 537–553. 10.1080/21505594.2020.1766790 32434419 PMC7250321

[B121] Young A. Stoilova-McPhie S. Rothnie A. Vallis Y. Harvey-Smith P. Ranson N. (2013). Hsc70-induced changes in clathrin-auxilin cage structure suggest a role for clathrin light chains in cage disassembly. *Traffic* 14 987–996. 10.1111/tra.12085 23710728 PMC3776051

[B122] Yu Y. Zhang Y. Chen X. Chen Y. (2019). Plant Noncoding RNAs: hidden players in development and stress responses. *Annu. Rev. Cell Dev. Biol.* 35 407–431. 10.1146/annurev-cellbio-100818-125218 31403819 PMC8034839

[B123] Yu Z. Li Q. Wang J. Yu Y. Wang Y. Zhou Q. (2020). Reactive oxygen species-related nanoparticle toxicity in the biomedical field. *Nanoscale Res. Lett.* 15 115. 10.1186/s11671-020-03344-7 32436107 PMC7239959

[B124] Yuan Y. Jiaoming L. Xiang W. Yanhui L. Shu J. Maling G. (2018). Analyzing the interactions of mRNAs, miRNAs, lncRNAs and circRNAs to predict competing endogenous RNA networks in glioblastoma. *J. Neurooncol.* 137 493–502. 10.1007/s11060-018-2757-0 29335913

[B125] Zang G. Zou H. Zhang Y. Xiang Z. Huang J. Luo L. (2016). The De-Etiolated 1 homolog of Arabidopsis modulates the ABA signaling pathway and ABA biosynthesis in rice. *Plant Physiol.* 171, 1259–1276. 10.1104/pp.16.00059 27208292 PMC4902595

[B126] Zhai R. Ye S. Zhu G. Lu Y. Ye J. Yu F. (2020). Identification and integrated analysis of glyphosate stress-responsive microRNAs, lncRNAs, and mRNAs in rice using genome-wide high-throughput sequencing. *BMC Genomics* 21:238. 10.1186/s12864-020-6637-6 32183693 PMC7076996

[B127] Zhang H. Wang Y. Wong J. J. Lim K. L. Liou Y. C. Wang H. (2014). Endocytic pathways downregulate the L1-type cell adhesion molecule neuroglian to promote dendrite pruning in *Drosophila*. *Dev. Cell* 30 463–478. 10.1016/j.devcel.2014.06.014 25158855

[B128] Zhang N. Wang Z. Bao Z. Yang L. Wu D. Shu X. (2018). MOS1 functions closely with TCP transcription factors to modulate immunity and cell cycle in *Arabidopsis*. *Plant J.* 93 66–78. 10.1111/tpj.13757 29086441

[B129] Zhao Z. Li Y. Zhao S. Zhang J. Zhang H. Fu B. (2018). Transcriptome analysis of gene expression patterns potentially associated with premature senescence in *Nicotiana tabacum* L. *Molecules* 23 2856. 10.3390/molecules23112856 30400189 PMC6278766

[B130] Zhou H. Liu Q. Li J. Jiang D. Zhou L. Wu P. (2012). Photoperiod- and thermo-sensitive genic male sterility in rice are caused by a point mutation in a novel noncoding RNA that produces a small RNA. *Cell Res.* 22 649–660. 10.1038/cr.2012.28 22349461 PMC3317565

[B131] Zhou Q. Yu Q. Wang Z. Pan Y. Lv W. Zhu L. (2013). Knockdown of GDCH gene reveals reactive oxygen species-induced leaf senescence in rice. *Plant Cell Environ.* 36 1476–1489. 10.1111/pce.12078 23421602

