# Supplementary material for: Whole-Transcriptome Analysis Reveals Autophagy Is Involved in Early Senescence of zj-es Mutant Rice
Source: Front Plant Sci. 2022 Jun 3;13:899054. doi: 10.3389/fpls.2022.899054 (PMC9204060; doi:10.3389/fpls.2022.899054)
Supplement: Supplementary file 1 [file Table_1.DOCX]

Supplementary Material


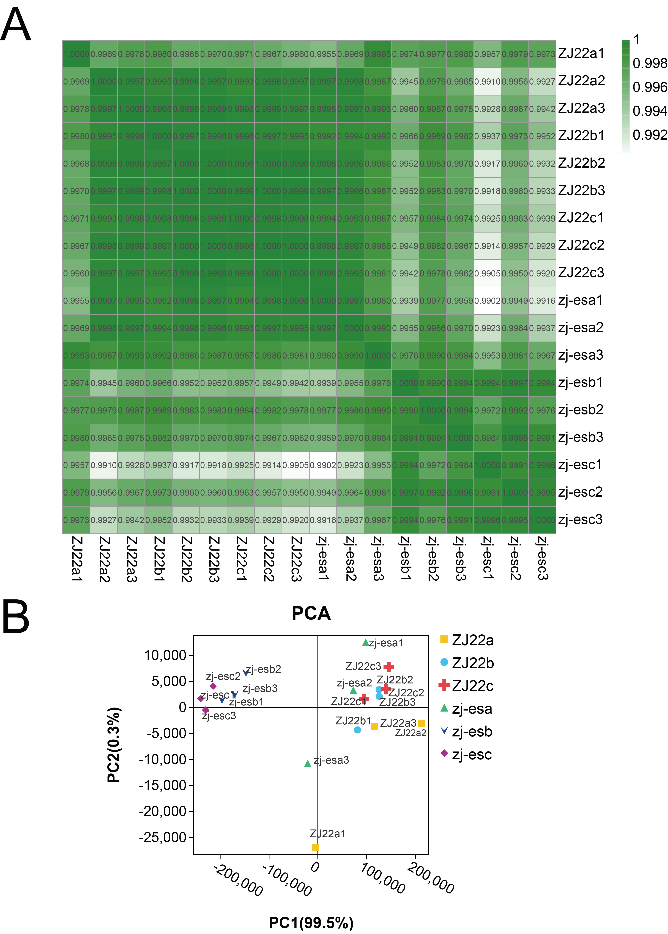


**Figure S1**. Correlation analysis among the samples including ZJ22a, ZJ22b, ZJ22c, *zj-es*a, *zj-es*b and *zj-es*c. (A) The correlation coefficient between each pair of samples showing the biological repeatability. The color intensity represents the p-value. (B) Principal component analysis (PCA) showing the correlation among samples at the level of two PCs.


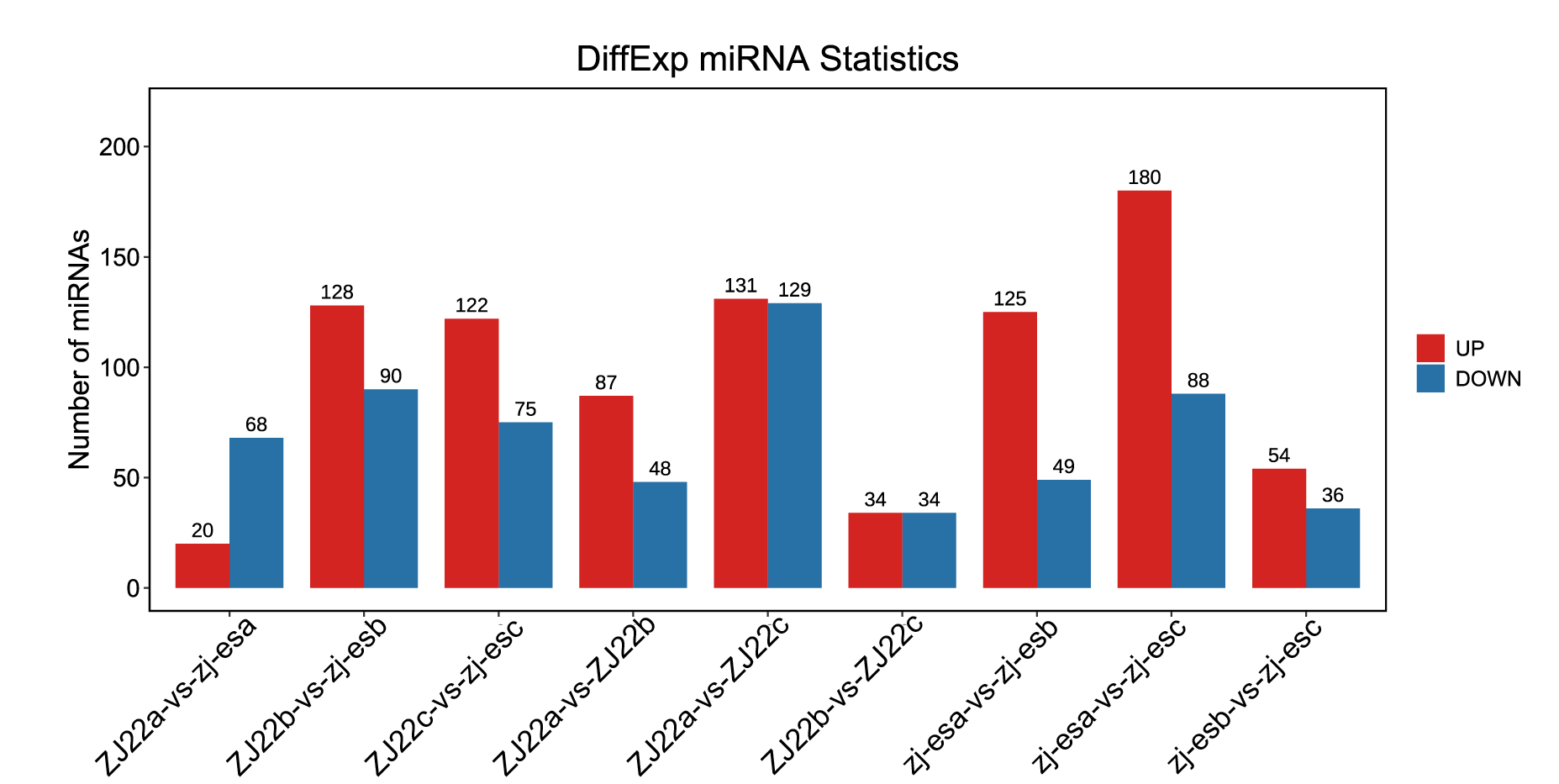


**Figure S2**. The number of significantly regulated genes of miRNA.


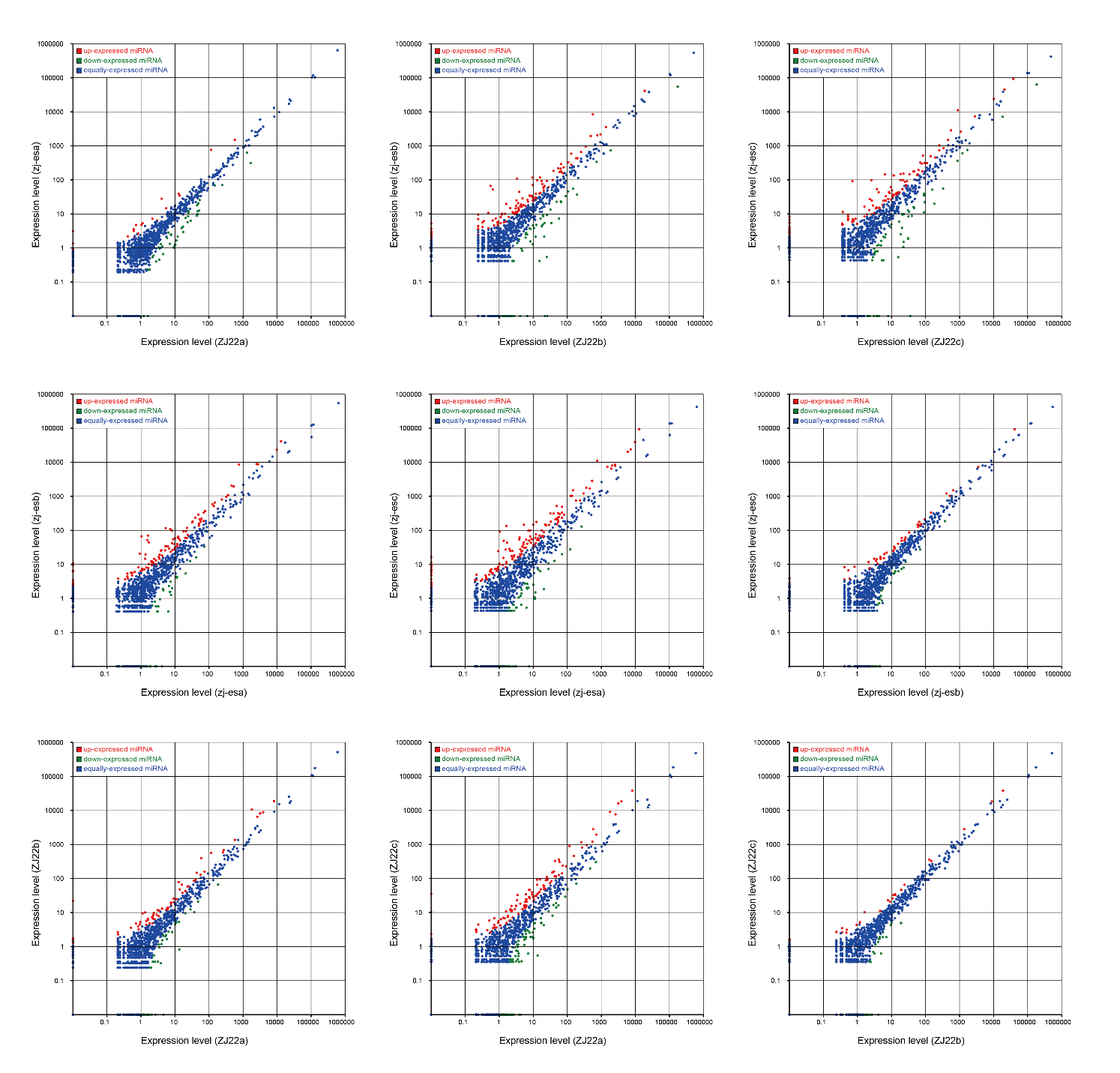


**Figure S3**. Scatter plot of miRNA for gene expression levels in each comparison group.


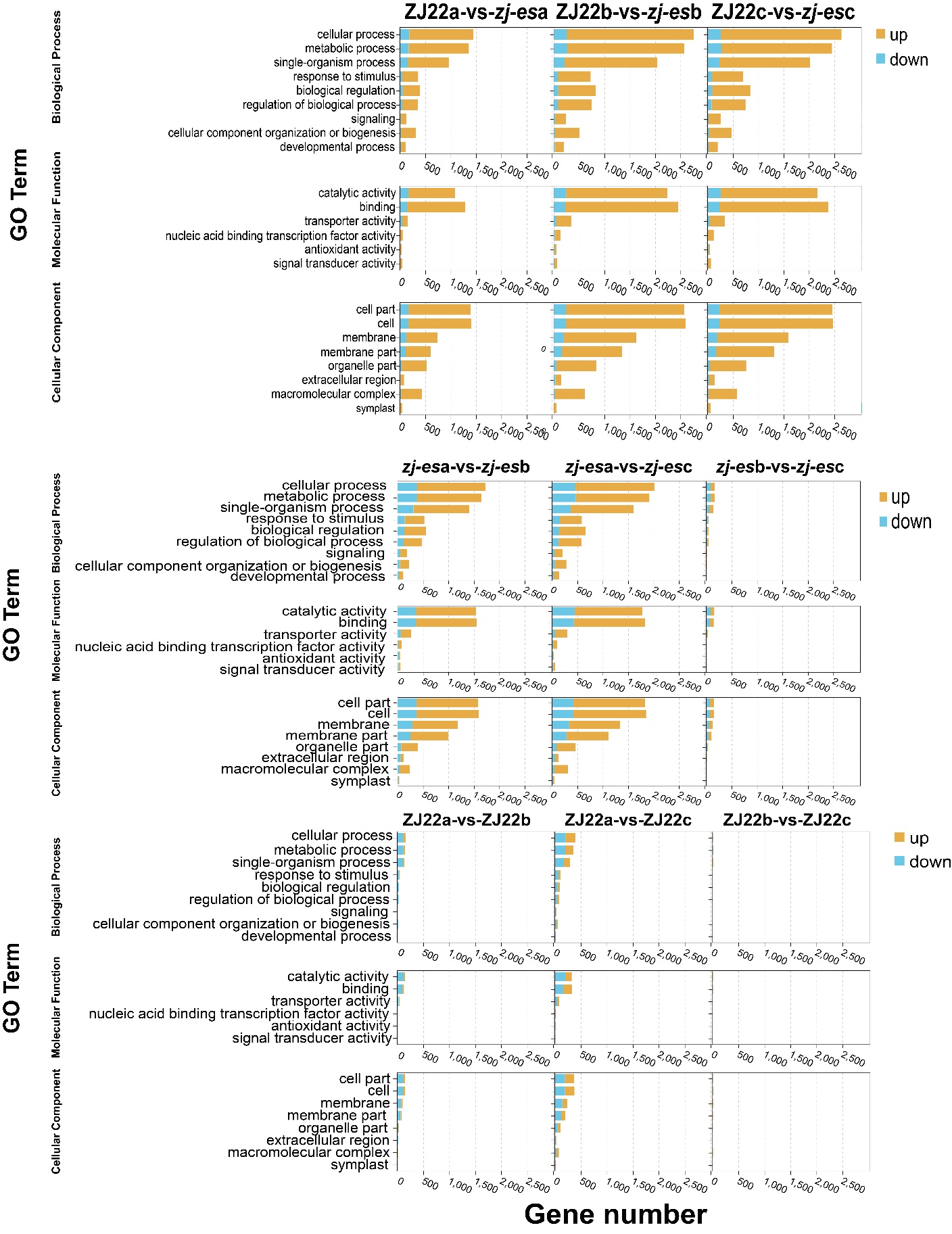


**Figure S4**. GO enrichment of DEmRNAs.


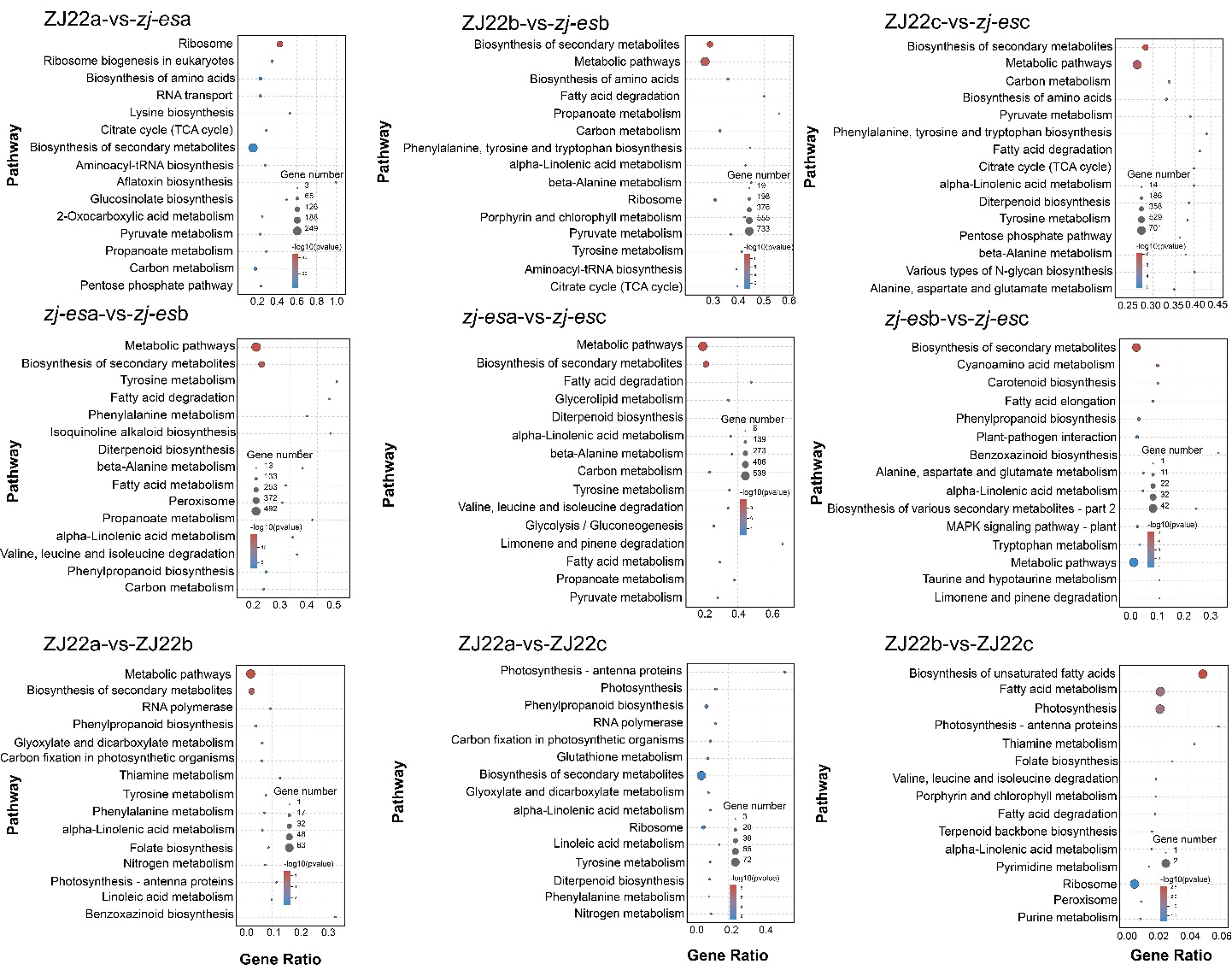


**Figure S5**. KEGG enrichment of DEmRNAs.


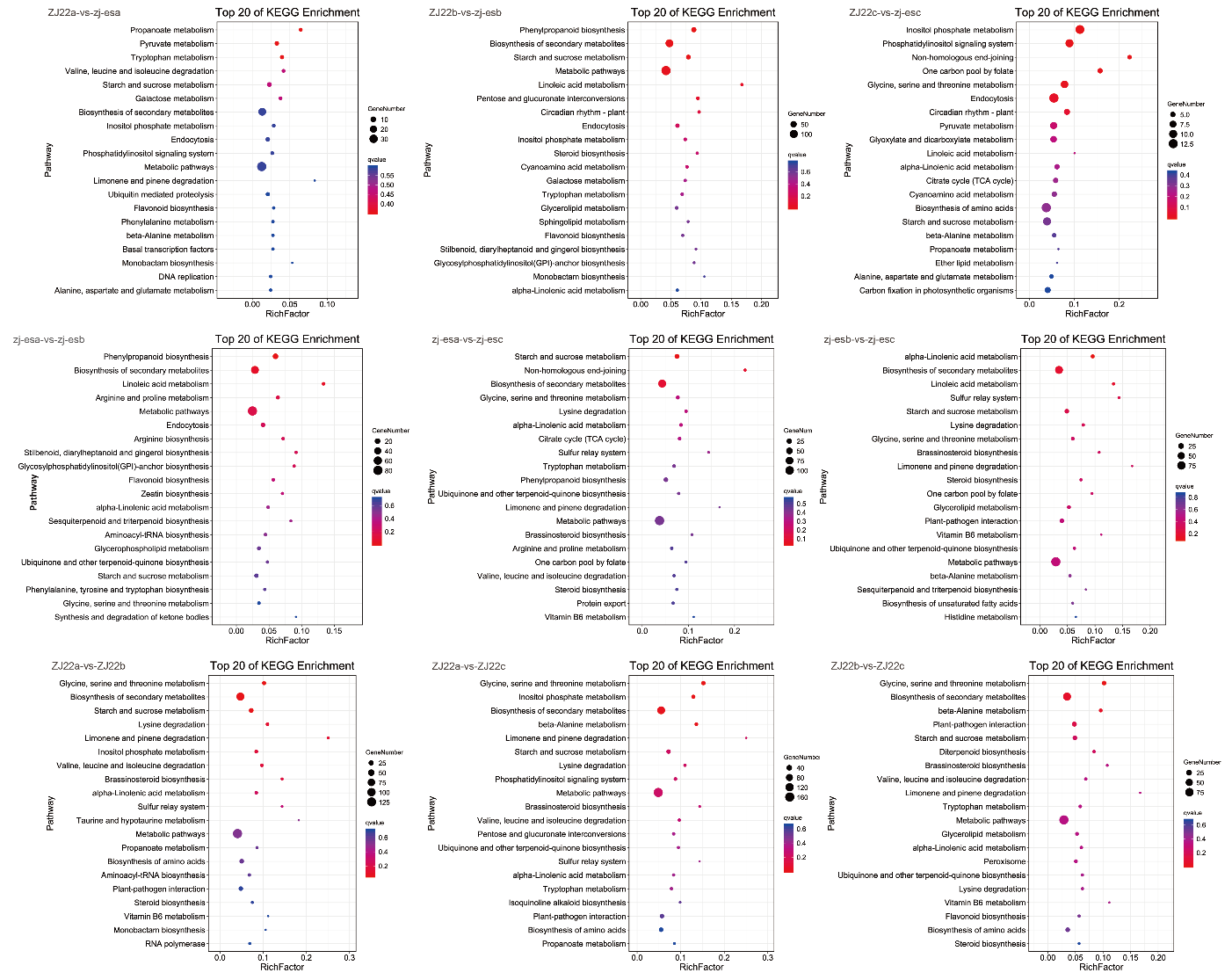


**Figure S6**. KEGG enrichment of target of DEmRNAs.


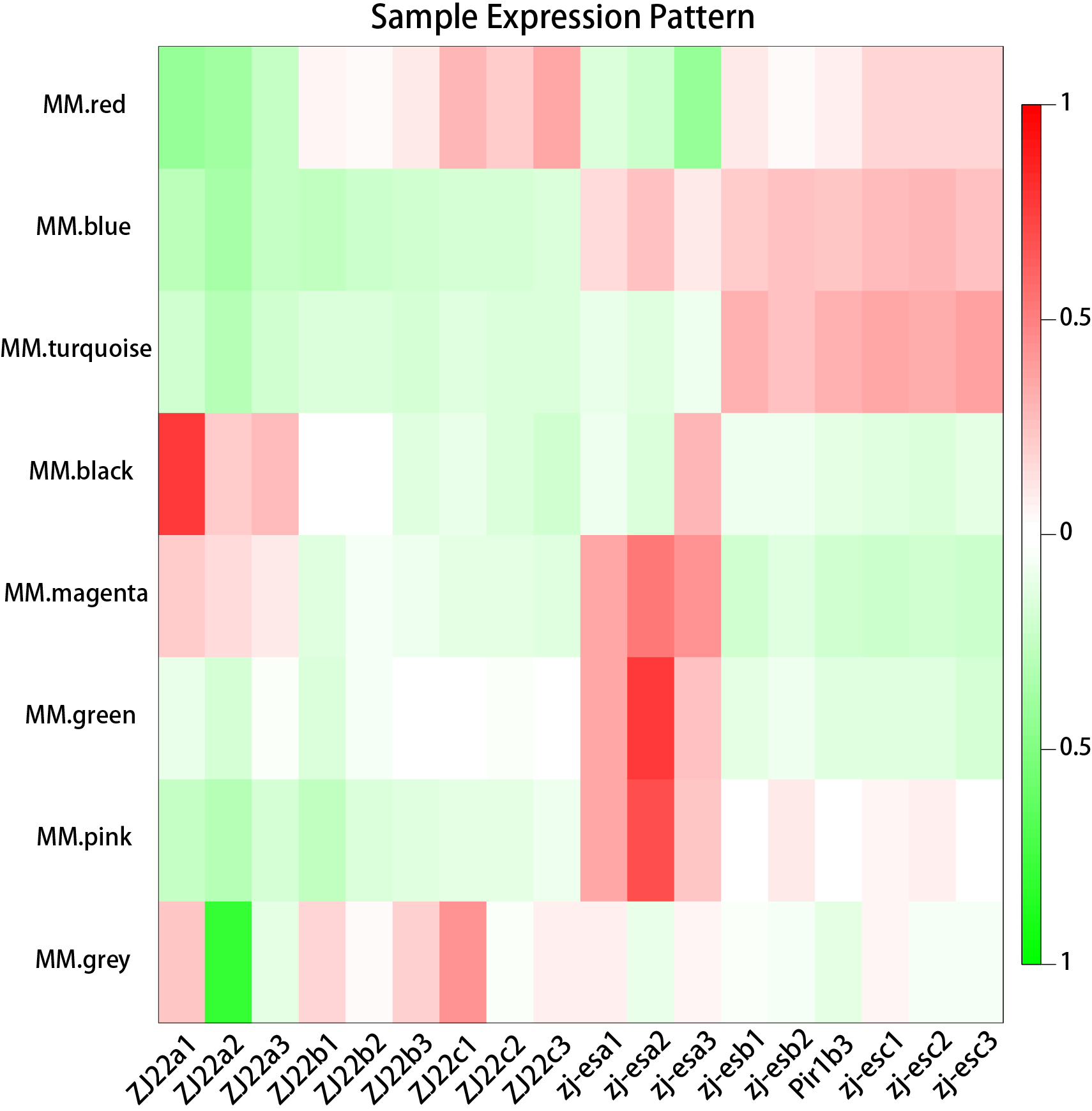


**Figure S7**. Sample expression pattern: heat map showing the sample expression patterns in the modules.


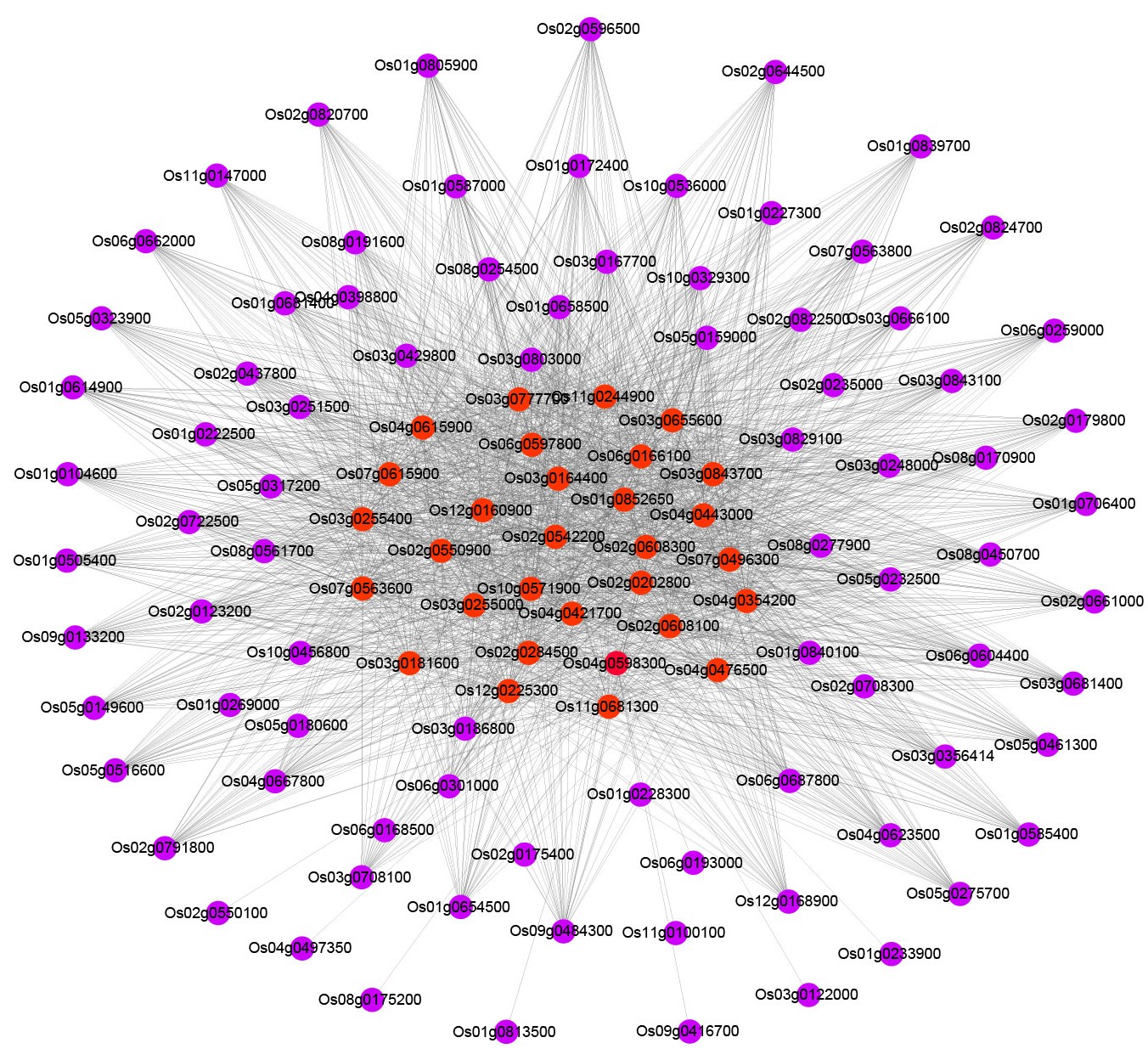


**Figure S8**. Regulatory network analysis of transcription factors-target genes. Red nodes represent transcription factors.


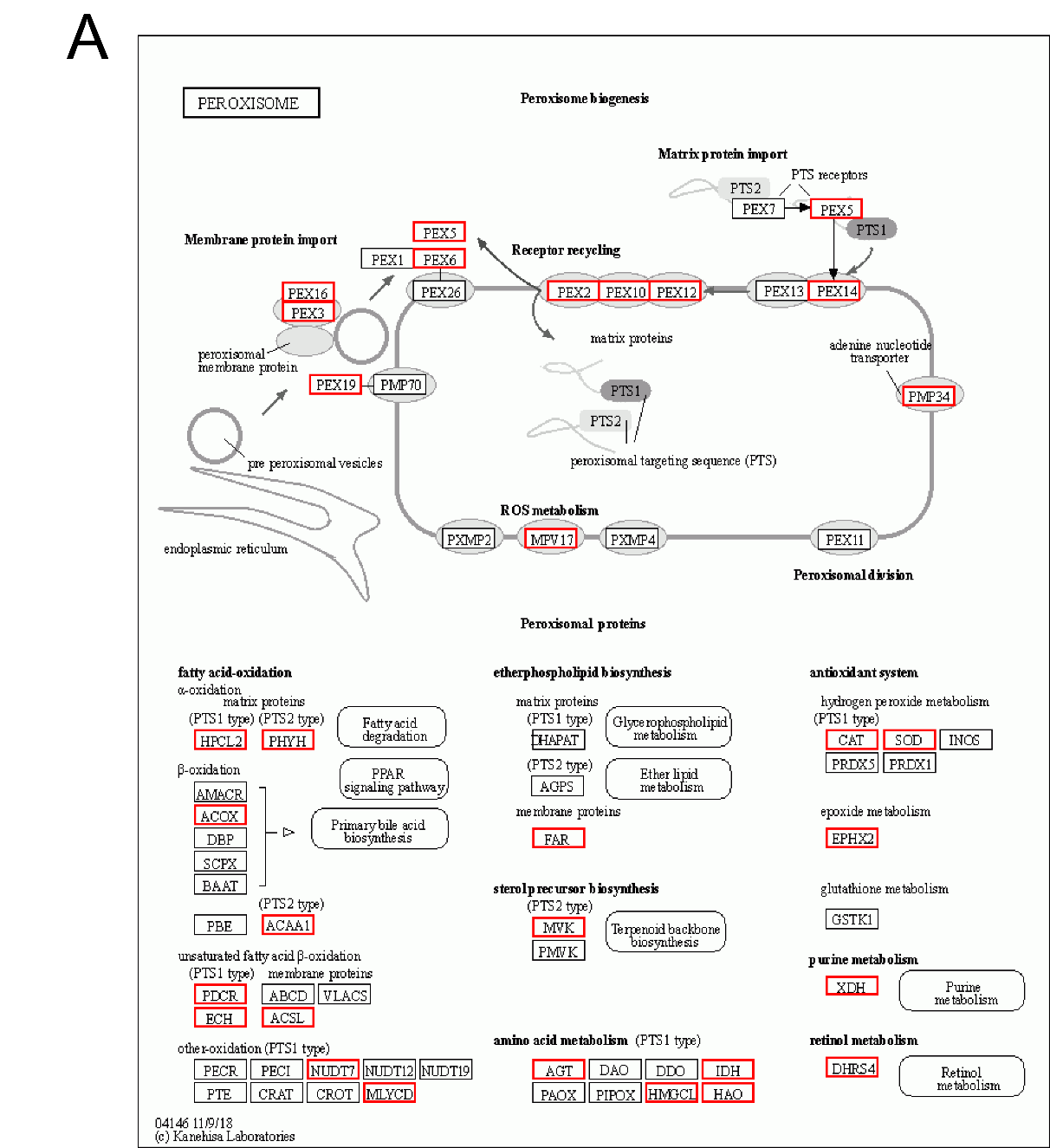


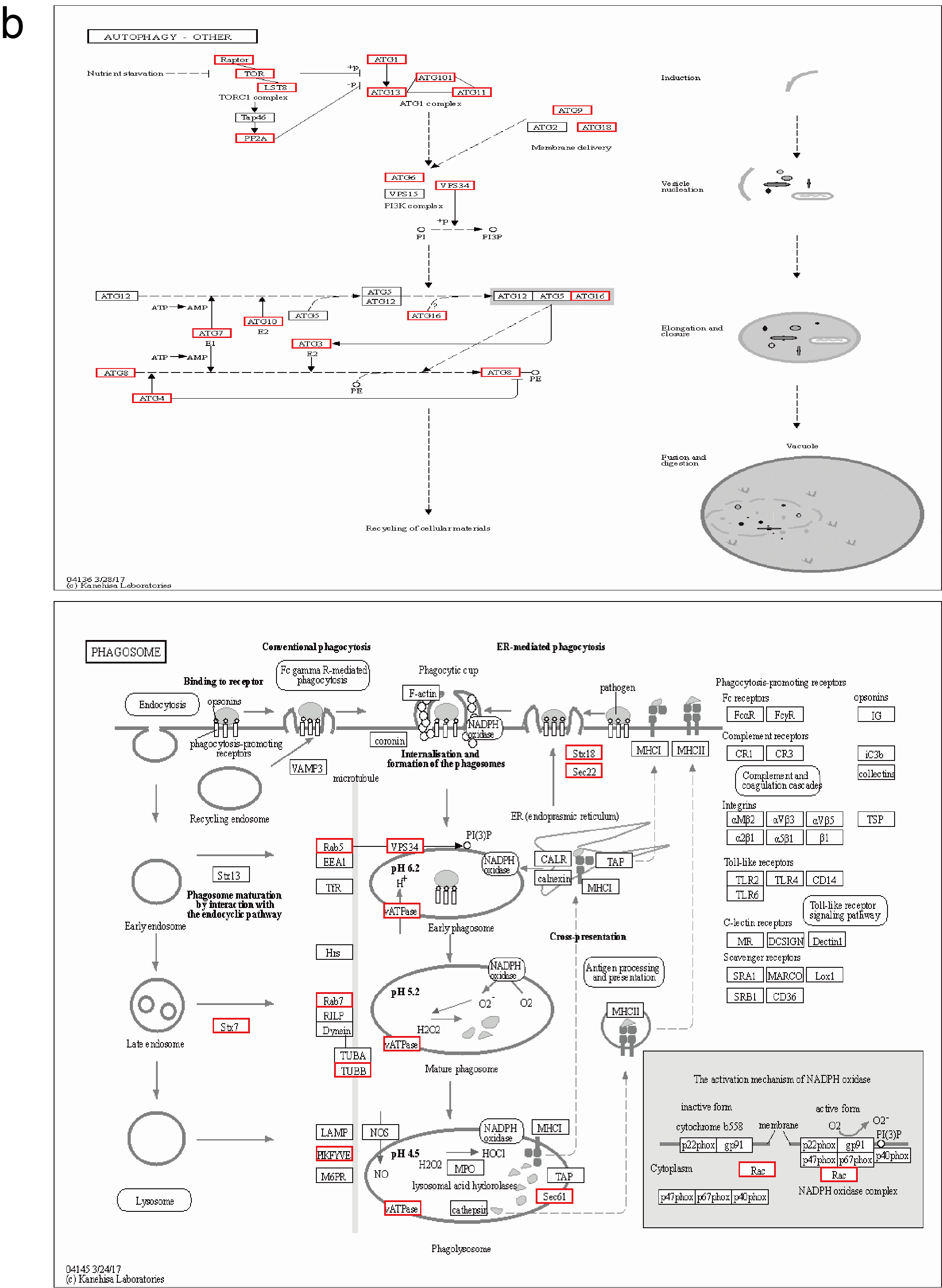


**B**


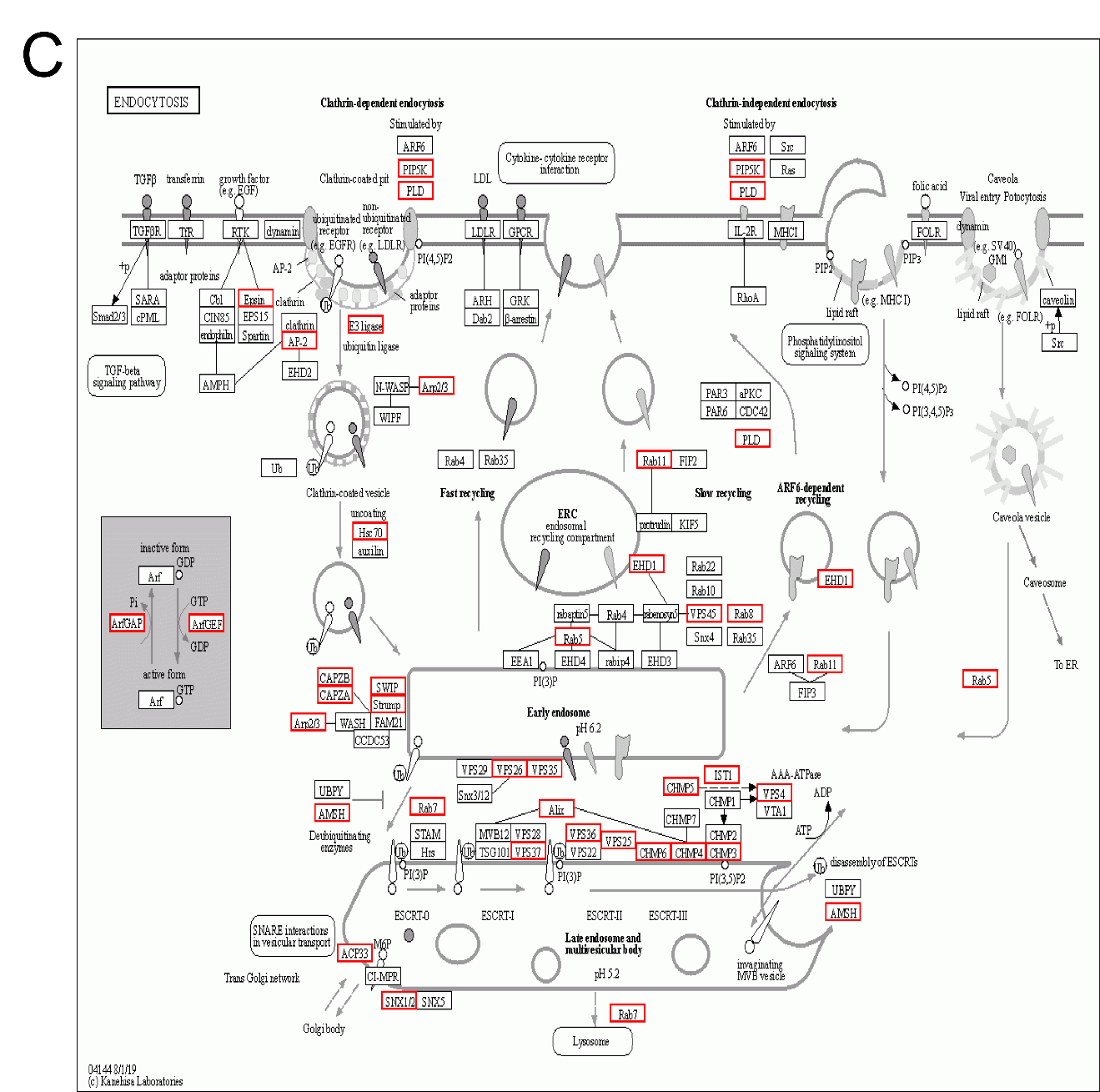


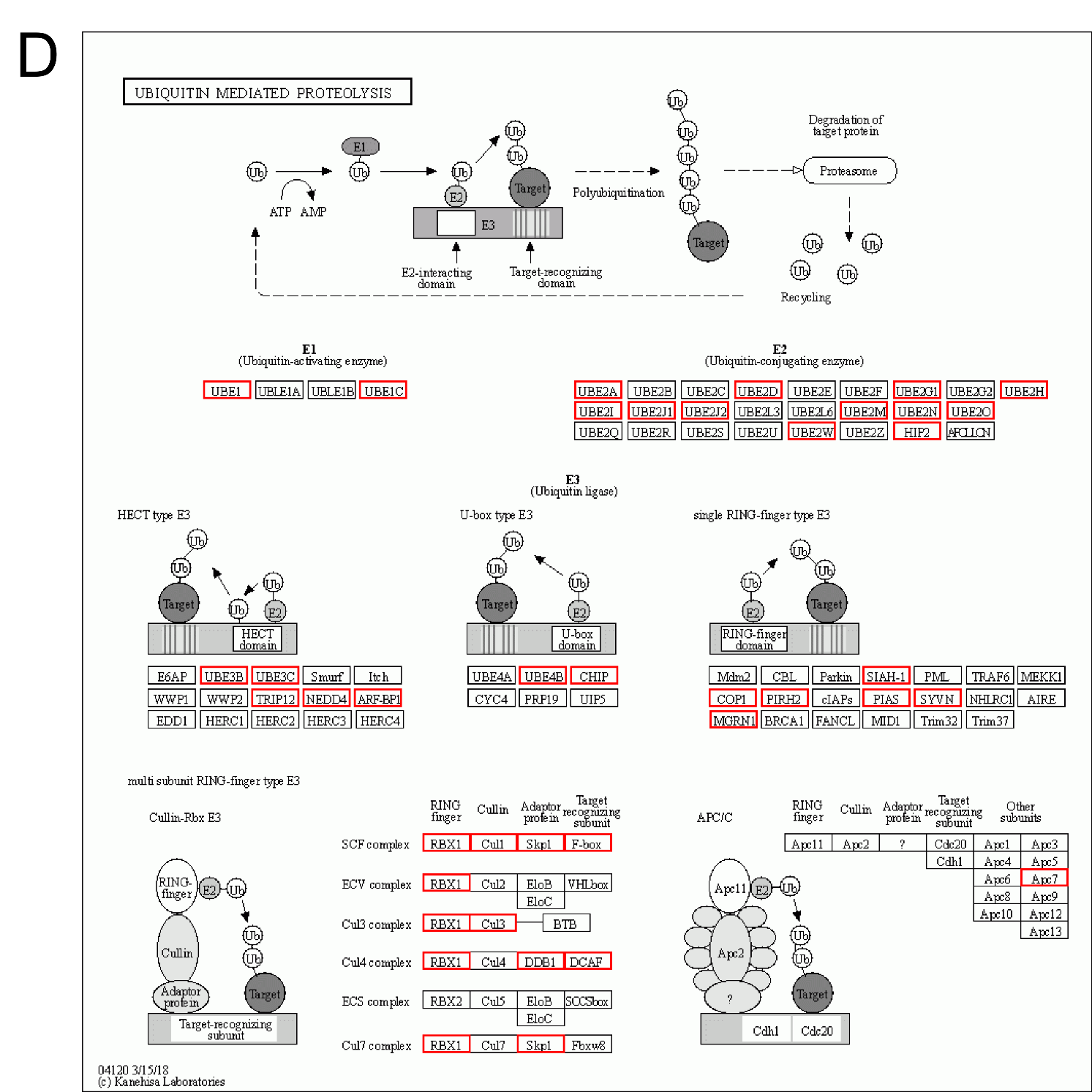


**Figure S9**. The pathways of peroxisomes (A); autophagy and phagosome (B); endocytosis (C); and ubiquitin mediated proteolysis (D) enriched by KEGG analysis. The KEGG pathway database is gained from Kanehisa laboratories.
