# Supplementary material for: Whole-Transcriptome Analysis Reveals Autophagy Is Involved in Early Senescence of zj-es Mutant Rice
Source: Front Plant Sci. 2022 Jun 3;13:899054. doi: 10.3389/fpls.2022.899054 (PMC9204060; doi:10.3389/fpls.2022.899054)
Supplement: Supplementary file 2 [file Table_2.DOCX]

Supplementary Material

**Table S1**. Genetic analysis of F_2_ populations

| Cross | Total number of plants | Number of normal plants | Number of plants  with early senescence | χ^2^(3:1) | χ^2^_0.05_ |
| --- | --- | --- | --- | --- | --- |
| ZJ22 × *zj-es* | 116 | 91 | 25 | 0.56 | 3.84 |
| 9311 × *zj-es* | 97 | 78 | 19 | 1.24 | 3.84 |
