# Supplementary material for: Whole-Transcriptome Analysis Reveals Autophagy Is Involved in Early Senescence of zj-es Mutant Rice
Source: Front Plant Sci. 2022 Jun 3;13:899054. doi: 10.3389/fpls.2022.899054 (PMC9204060; doi:10.3389/fpls.2022.899054)
Supplement: Supplementary file 4 [file Table_9.DOCX]

Table S4. Overview mRNA-sequencing reads generated from each sample.

| Sample | Raw Reads_count | Clean Reads_count (%) | Total Mapped Reads_count | Mapped% | Uniquely Mapped Reads_count | Uniquely Mapped% |
| --- | --- | --- | --- | --- | --- | --- |
| ZJ22a1 | 82571648 | 82137562(99.47%) | 75154882 | 93.54% | 31695487 | 39.45% |
| ZJ22a2 | 83499622 | 83131956(99.56%) | 79025025 | 95.80% | 22914589 | 27.78% |
| ZJ22a3 | 83377144 | 83025694(99.58%) | 75932763 | 92.84% | 26897755 | 32.89% |
| ZJ22b1 | 73309386 | 72876406(99.41%) | 64486263 | 94.75% | 25595877 | 37.61% |
| ZJ22b2 | 82319516 | 81979600(99.59%) | 77852924 | 95.44% | 28700347 | 35.18% |
| ZJ22b3 | 82536100 | 82109128(99.48%) | 76494534 | 94.69% | 29801916 | 36.89% |
| ZJ22c1 | 81908742 | 81428070(99.41%) | 76281101 | 94.30% | 30172380 | 37.30% |
| ZJ22c2 | 83989850 | 83637258(99.58%) | 79874803 | 96.02% | 27772336 | 33.39% |
| ZJ22c3 | 87527500 | 87163236(99.58%) | 81980002 | 94.69% | 26781641 | 30.93% |
| *zj-es*a1 | 87068088 | 86530220(99.38%) | 81712590 | 94.86% | 34397370 | 39.93% |
| *zj-es*a2 | 86786702 | 86164960(99.28%) | 80680725 | 94.17% | 35317988 | 41.22% |
| *zj-es*a3 | 75158288 | 74626418(99.29%) | 69108729 | 93.19% | 34604806 | 46.66% |
| *zj-es*b1 | 79098554 | 78568226(99.33%) | 73215778 | 93.80% | 47242842 | 60.53% |
| *zj-es*b2 | 81818766 | 81333724(99.41%) | 75676280 | 93.89% | 46961544 | 58.26% |
| *zj-es*b3 | 78014288 | 77648944(99.53%) | 73161456 | 94.67% | 44381045 | 57.43% |
| *zj-es*c1 | 82289798 | 81921344(99.55%) | 76348923 | 94.89% | 50302075 | 62.52% |
| *zj-es*c2 | 78124444 | 77980850(99.82%) | 73258433 | 96.56% | 46144656 | 60.82% |
| *zj-es*c3 | 83842898 | 83438860(99.52%) | 75432804 | 91.64% | 48982518 | 59.51% |

Table S6. Expression of lncRNA in each sample

| Sample | Refer_lncs | sequenced_Refer_lncs(%) | Novel_lncs | sequenced_Novel_lncs(%) | Total_lncs | sequenced_Total_lncs(%) |
| --- | --- | --- | --- | --- | --- | --- |
| all | 95 | 50 (52.63%) | 5194 | 5194 (100.00%) | 5289 | 5244 (99.15%) |
| ZJ22a1 | 95 | 16 (16.84%) | 5194 | 3111 (59.90%) | 5289 | 3127 (59.12%) |
| ZJ22a2 | 95 | 16 (16.84%) | 5194 | 2479 (47.73%) | 5289 | 2495 (47.17%) |
| ZJ22a3 | 95 | 18 (18.95%) | 5194 | 3002 (57.80%) | 5289 | 3020 (57.10%) |
| ZJ22b1 | 95 | 27 (28.42%) | 5194 | 3065 (59.01%) | 5289 | 3092 (58.46%) |
| ZJ22b2 | 95 | 32 (33.68%) | 5194 | 3198 (61.57%) | 5289 | 3230 (61.07%) |
| ZJ22b3 | 95 | 20 (21.05%) | 5194 | 3052 (58.76%) | 5289 | 3072 (58.08%) |
| ZJ22c1 | 95 | 29 (30.53%) | 5194 | 3080 (59.30%) | 5289 | 3109 (58.78%) |
| ZJ22c2 | 95 | 29 (30.53%) | 5194 | 2929 (56.39%) | 5289 | 2958 (55.93%) |
| ZJ22c3 | 95 | 16 (16.84%) | 5194 | 3022 (58.18%) | 5289 | 3038 (57.44%) |
| zj-esa1 | 95 | 16 (16.84%) | 5194 | 3282 (63.19%) | 5289 | 3298 (62.36%) |
| zj-esa2 | 95 | 24 (25.26%) | 5194 | 3491 (67.21%) | 5289 | 3515 (66.46%) |
| zj-esa3 | 95 | 22 (23.16%) | 5194 | 3549 (68.33%) | 5289 | 3571 (67.52%) |
| zj-esb1 | 95 | 37 (38.95%) | 5194 | 3675 (70.75%) | 5289 | 3712 (70.18%) |
| zj-esb2 | 95 | 27 (28.42%) | 5194 | 3722 (71.66%) | 5289 | 3749 (70.88%) |
| zj-esb3 | 95 | 20 (21.05%) | 5194 | 3743 (72.06%) | 5289 | 3763 (71.15%) |
| zj-esc1 | 95 | 25 (26.32%) | 5194 | 3713 (71.49%) | 5289 | 3738 (70.67%) |
| zj-esc2 | 95 | 35 (36.84%) | 5194 | 3741 (72.03%) | 5289 | 3776 (71.39%) |
| zj-esc3 | 95 | 29 (30.53%) | 5194 | 3738 (71.97%) | 5289 | 3767 (71.22%) |

Table S7. Identification of miRNA in each sample.

| Sample | exist_mirna_num | known_mirna_num | novel_mirna_num |
| --- | --- | --- | --- |
| all | 495 | 555 | 561 |
| ZJ22a1 | 375 | 229 | 470 |
| ZJ22a2 | 359 | 209 | 415 |
| ZJ22a3 | 366 | 250 | 419 |
| ZJ22b1 | 379 | 233 | 355 |
| ZJ22b2 | 347 | 233 | 330 |
| ZJ22b3 | 362 | 229 | 405 |
| ZJ22c1 | 368 | 204 | 312 |
| ZJ22c2 | 354 | 215 | 248 |
| ZJ22c3 | 353 | 224 | 312 |
| zj-esa1 | 361 | 224 | 417 |
| zj-esa2 | 369 | 249 | 379 |
| zj-esa3 | 377 | 222 | 488 |
| zj-esb1 | 339 | 200 | 369 |
| zj-esb2 | 364 | 230 | 392 |
| zj-esb3 | 354 | 199 | 345 |
| zj-esc1 | 343 | 198 | 277 |
| zj-esc2 | 355 | 186 | 326 |
| zj-esc3 | 364 | 237 | 318 |
