# Supplementary material for: Whole-Transcriptome Analysis Reveals Autophagy Is Involved in Early Senescence of zj-es Mutant Rice
Source: Front Plant Sci. 2022 Jun 3;13:899054. doi: 10.3389/fpls.2022.899054 (PMC9204060; doi:10.3389/fpls.2022.899054)
Supplement: Supplementary file 14 [file Table_15.DOCX]

**Table S18.** Molecular markers with polymorphism between *zj-es* and 9311.

| **Marker** | **Chromosome** | **Position (cM)** | **Type** | **Forward Primer** | **Reverse Primer** |
| --- | --- | --- | --- | --- | --- |
| RM338 | 3 | 50.9 | SSR | CACAGGAGCAGGAGAAGAGC | GGCAAACCGATCACTCAGTC |
| RM156 | 3 | 60.1 | SSR | GCCGCACCCTCACTCCCTCCTC | TCTTGCCGGAGCGCTTGAGGTG |
| RM16 | 3 | 89.0 | SSR | CGCTAGGGCAGCATCTAAA | AACACAGCAGGTACGCGC |
| RM168 | 3 | 108.1 | SSR | TGCTGCTTGCCTGCTTCCTTT | GAAACGAATCAATCCACGGC |
| RM186 | 3 | 110.8 | SSR | TCCTCCATCTCCTCCGCTCCCG | GGGCGTGGTGGCCTTCTTCGTC |
| RM15882 | 3 | 116.3 | SSR | CCACCTCAACCACCACTTCATCG | TTGTCGCCTCCCTCAACTCAACC |
| RM3525 | 3 | 116.9 | SSR | ACACTCTCAGCTCATCAAGACC | GGGCAAGTGGTCAAATCTTG |
| RM5172 | 3 | 117.0 | SSR | ATATGCATGCGTTTATTACC | TGGCTGTTATGTGAAATACA |
| RM5992 | 3 | 117.4 | SSR | GGTAGGTTTTTGGCCGAATC | AGAGAGGAGAATCGCCGC |
| RM3829 | 3 | 118.6 | SSR | AAGTAGAGGCGGAGAGCGAG | TCAGCCTCTCAGGAATCTGC |
| RM5813 | 3 | 119.2 | SSR | GCAGCCCTAGCAATTCAGTC | CTCCCTTTCCCTCCACCAC |
| RM416 | 3 | 120.2 | SSR | GGGAGTTAGGGTTTTGGAGC | TCCAGTTTCACACTGCTTCG |
| RM448 | 3 | 120.8 | SSR | TCTGATCTTGATGCAGGCAC | TCTCCCGATTTGGACAGATC |
| RM293 | 3 | 121.8 | SSR | TCGTTGGGAGGTATGGTACC | CTTTATCTGATCCTTGGGAAGG |
| RM468 | 3 | 125.7 | SSR | CCCTTCCTTGTTGTGGCTAC | TGATTTCTGAGAGCCAACCC |
| RM422 | 3 | 129.7 | SSR | TTCAACCTGCATCCGCTC | CCATCCAAATCAGCAACAGC |
| RM6712 | 3 | 135.5 | SSR | GCGCATCATCACTTCATCAG | AGATGAGCCTATCAGCTGCC |
| RM85 | 3 | 139.8 | SSR | CCAAAGATGAAACCTGGATTG | GCACAAGGTGAGCAGTCC |
