# Supplementary material for: Whole-Transcriptome Analysis Reveals Autophagy Is Involved in Early Senescence of zj-es Mutant Rice
Source: Front Plant Sci. 2022 Jun 3;13:899054. doi: 10.3389/fpls.2022.899054 (PMC9204060; doi:10.3389/fpls.2022.899054)
Supplement: Supplementary file 15 [file Table_14.DOCX]

#Take sample ZJ22a1 for example

# 数据过滤

fastp -a AGATCGGAAGAGC -q 20 -u 50 -n 15 -l 50 -w 1 -i 1.rawData/ZJ22a1_1.fq.gz -I 1.rawData/ZJ22a1_2.fq.gz -o 2.filterData/ZJ22a1_1.fq.gz -O 2.filterData/ZJ22a1_2.fq.gz -j 2.filterData/ZJ22a1.json -h 2.filterData/ZJ22a1.html

# 去rRNA(使用数据过滤后的fq文件)

bowtie2 --local -S /dev/null -p 4 --mm -x <rRNA index> -1 ZJ22a1_1.fq.gz -2 ZJ22a1_2.fq.gz --un-conc-gz ZJ22a1_%.fq.gz 2>ZJ22a1.rRNA.log

# 比对基因组(使用去rRNA后的fq文件)

hisat2 --rna-strandness RF -p 4 -q -x <genome index> -1 ZJ22a1_1.fq.gz -2 ZJ22a1_2.fq.gz --known-splicesite-infile <splices sites file> -t --dta --summary-file ZJ22a1.stat --new-summary

# 重构转录本

stringtie --rf -f 0.3 ZJ22a1.sort.bam -G ref.gtf -p 1 -o ZJ22a1.gtf

# merge转录本(assembly_GTF.list中文件内容为上一步重构转录本生成的所有样本的gtf文件名)

stringtie --merge -f 0.3 -m 200 -G ref.gtf -o merged.gtf assembly_GTF.list

# LncRNA鉴定

##CPC2软件预测新转录本的编码能力(newT.fa为新转录本的fasta格式文件，转录本长度≥200bp且exon数目≥1)

python CPC2-beta/bin/CPC2.py -i newT.fa -o CPC2.results

##CNCI软件预测新转录本的编码能力(newT.fa为新转录本的fasta格式文件，转录本长度≥200bp且exon数目≥1)

python CNCI_package/CNCI.py -f newT.fa -o results -p 3 -m pl

##最后取以上两个软件没有编码潜能的转录本交集作为可靠的LncRNA预测结果

# 定量(使用去rRNA后的fq文件)

##比对所有外显子序列

bowtie2 --fr --nofw -k 200 --no-mixed --no-discordant --gbar 99999999 --dpad 0 --mp 1,1 --np 1 --score-min L,0,-0.1 -q -X 800 --phred33-quals -x <exon index> -1 <(zcat ZJ22a1_1.fq.gz) -2 <(zcat ZJ22a1_2.fq.gz) -p 4

##RSEM定量

rsem-calculate-expression --paired-end -p 4 --no-bam-output --bam ZJ22a1.bowtie2.bam <exon RSEM index> ZJ22a1
